# Isolation of phytochemicals and exploration the mechanism of *Dolichandrone spathacea* in the treatment of chronic bronchitis by integrating network pharmacology, molecular docking, and experimental validation

**DOI:** 10.1186/s40529-025-00464-0

**Published:** 2025-08-11

**Authors:** Dang-Khoa Nguyen, Ta-Wei Liu, Man-Hsiu Chu, Quoc-Dung Tran Huynh, Truc-Ly Thi Duong, Thuy-Tien Thi Phan, Duyen Thi My Huynh, Yun-Han Wang, Shu-Mei Wang, Su-Jung Hsu, Ching-Kuo Lee

**Affiliations:** 1 School of Pharmacy, College of Pharmacy, Taipei Medical University, Taipei, 11031 Taiwan; 2 School of Pharmacy, University of Medicine and Pharmacy at Ho Chi Minh City, Ho Chi Minh City, 700000 Vietnam; 3 Program in Clinical Drug Development of Herbal Medicine, College of Pharmacy, Taipei Medical University, Taipei, 11031 Taiwan; 4 Institute of Pharmaceutical Education and Research, Binh Duong University, Binh Duong, 820000 Vietnam; 5 Faculty of Traditional Medicine, Can Tho University of Medicine and Pharmacy, Can Tho, 900000 Vietnam; 6 Graduate Institute of Biomedical Materials and Tissue Engineering, College of Biomedical Engineering, Taipei Medical University, Taipei, 11031 Taiwan; 7 Department of Pharmaceutical and Pharmaceutical Technology, Faculty of Pharmacy, Can Tho University of Medicine and Pharmacy, Can Tho, 900000 Vietnam; 8 Program in Drug Discovery and Development Industry, College of Pharmacy, Taipei Medical University, Taipei, 11031 Taiwan; 9 Institute of Fisheries Science, National Taiwan University, Taipei City, Taiwan

**Keywords:** Chronic bronchitis, Docking, Isolation, Network pharmacology, Validation

## Abstract

**Background:**

*Dolichandrone spathacea* (*D. spathacea*) is a traditional medicine used to treat chronic bronchitis (CB) in Vietnam and India. However, phytochemicals and potential mechanisms of this species against CB have not been fully illuminated. Therefore, this study aimed to isolate and elucidate the phytochemicals of *D. spathacea*, clarify its potential molecular mechanisms and key therapeutic targets in treating CB through network pharmacology and validate these findings using molecular docking, and experimental approaches.

**Results:**

Three compounds, beta-sitosterol, 6-O-*trans*-*p-*coumaroyl ajugol, 6-*O*-[(*E*)-4-methoxycinnamoyl] catalpol were isolated from the EtOAc fraction, with beta-sitosterol being reported for the first time of this species. After combining the phytochemicals of this species identified in this study with those reported in the literature references, 59 compounds were obtained, and 30 bioactive compounds were screened. Among these, luteolin was predicted to interact with the highest number of CB-related proteins. Using the GeneCards and DrugBank databases, 66 intersecting target genes were identified between *D. spathacea* and CB. The protein–protein interaction analysis identified core targets, including TNF, AKT1, SRC, EGFR, IL2, MMP-9, HSP90AA1, and PTGS2. The KEGG enrichment analyses suggested that this species exerts its therapeutic effects on CB by modulating various biological processes and pathways*.* Notably, the top three target genes—PTGS2, TNF, and MMP-9—were enriched in the TNF and IL-17 signaling pathways. The computational docking suggested that PTGS2, TNF, and MMP-9 could bind to all key bioactive compounds of *D. spathacea*. The experimental validation revealed that ethanol extract inhibited nitric oxide production induced by LPS, with an IC_50_ value of 25.34 μg/mL. At the concentration of 100 μg/mL, the ethanol extract effectively inhibited the production of TNF-α, IL-1β cytokine, with inhibition rates of 71.67%, and 90.22%, respectively.

**Conclusion:**

This study systematically investigated the phytoconstituents, core target genes, and key mechanisms of *D. spathacea* in the treatment of chronic bronchitis. It highlights the role of this species in modulating the TNF and IL-17 signaling pathways in CB therapy. The findings suggest that *D. spathacea* exhibits significant anti-inflammatory effects on CB, providing robust scientific evidence and novel insights for further research on chronic bronchitis.

**Supplementary Information:**

The online version contains supplementary material available at 10.1186/s40529-025-00464-0.

## Background

Chronic bronchitis is characterized by a persistent cough with mucus production lasting over three months for at least three months in two consecutive years. It is often associated with smoking and commonly coexists with chronic obstructive pulmonary disease (COPD). It's a variable condition within COPD, carrying significant clinical implications, such as accelerated lung function decline, an increased risk of airflow obstruction in smokers, heightened susceptibility to respiratory infections, more frequent exacerbations, and an elevated risk of mortality. Excessive mucus production by goblet cells is the primary cause, leading to airway obstruction, structural changes, and increased airway collapsibility. Chronic inflammation causes enlargement of mucus glands and an increase in goblet cells in the airways. In cases of COPD accompanied by chronic bronchitis, macrophages are believed to play a crucial role in orchestrating the inflammatory response (Barnes 2016; Corhay et al. 2013; Kim and Criner 2013; Mejza et al. 2017). The primary goal of managing chronic bronchitis is to alleviate symptoms, prevent complications, and slow disease progression, which can be achieved through medical and non-medical strategies. Pharmaceuticals play a crucial role, with bronchodilators helping to widen airways and facilitate mucus clearance, while glucocorticoids reduce inflammation and mucus production, although their use requires caution due to potential side effects. Antibiotics are generally not recommended, but macrolide therapy, with its anti-inflammatory properties, may be considered. Additionally, phosphodiesterase-4 inhibitors help reduce inflammation and relax smooth muscles in airways by preventing the breakdown of cyclic adenosine monophosphate, which can trigger the release of inflammatory substances (Arkhipov et al. 2017; Perotin et al. 2018; Song et al. 2018).

*Dolichandrone spathacea*, a member of the Bignoniaceae family, is commonly found in the natural habitats along riverbanks and mangrove ecosystems across the Asia–Pacific region. Traditionally, its fruits and flowers have been used to alleviate symptoms of cholera, haematemesis, diuresis, and chlorosis. The bark has been utilized as a fever reducer for illnesses like vomiting, diarrhea, asthma, and inflammation. The leaves are used to treat thrush and as a laxative, and they are being researched for their antiseptic properties and potential antitumor effects; stems are traditionally used to alleviate chronic bronchitis (Jackes 2017; Nguyen et al. 2018a, b). The studies of this species have identified several key phytochemicals, including steroids, flavonoids, saponins, iridoids, phenolics, triterpenoids, and phenylethanoid glycosides. These compounds are the primary bioactive compounds responsible for the plant's medicinal properties (Nguyen et al. 2024; Thao et al. 2021b). The pharmacological studies have demonstrated that *D. spathacea* exhibits anti-hyperglycemic effects, antioxidant, anti-xanthine oxidase properties, and antibacterial activity (Kaewpiboon et al. 2012; Nguyen et al. 2018b, 2024).

Traditional Chinese Medicine (TCM) has profoundly influenced the field of network pharmacology, which combines diverse research methods to study drug interactions with biological systems. Rooted in cultural traditions and holistic theories of syndrome differentiation and treatment, TCM is supported by extensive clinical experience. Many TCM formulations provide the foundation for comprehensive treatment strategies. Network pharmacology integrates systems and reductionist theories, macroscopic and microscopic investigations, both in vitro and in vivo studies to understand these interactions comprehensively. Advances in systems biology, network biology, and chemical biology have propelled this field in drug discovery. By merging reductionist and systems-based methodologies with computational and experimental approaches, network pharmacology transitions from the conventional "one target, one drug" paradigm to a "network target, multicomponent therapeutics" model, aligning with TCM's holistic principles. This approach is poised to revolutionize drug research by identifying bioactive compounds and their molecular targets from various herbs and formulations (Li et al. 2021, 2014; Su et al. 2021).

The study aimed to identify some phytoconstituents of *D. spathacea* by isolating and elucidating the structures of specific compounds using nuclear magnetic resonance and mass spectrometry. Network pharmacology was employed to explore the potential mechanisms in treating chronic bronchitis of *D. spathacea*. Molecular docking was used to assess the binding efficacy of these bioactive compounds to target proteins associated with this disease. Furthermore, the study validated the anti-inflammatory activities of the ethanol extract using RAW 264.7 cells.

## Methods

### Materials

The dry material was obtained from the Hospital of Traditional Medicine, Ho Chi Minh City, Vietnam. The sample was identified using the rbcL gene region amplification, sequenced via the Sanger method, and aligned to the NCBI database using BLAST. These procedures were performed by the DNA Sequencing Limited Liability Company in Can Tho City, Vietnam (Fig. S1). The samples were subsequently investigated at the Institute of Pharmacognosy, Taipei Medical University, Taiwan.

### General experimental procedures

Both reverse and normal phase column chromatographies were used to isolate and purify compounds. The semi-preparative liquid column, the Purospher® STAR RP-18 end-capped (5 µm), was used for the reverse phase, while a semi-preparative liquid column, a Phenomenex® Luna 5 μm Silica 100 Å, was used for the normal phase. Detection was performed using infrared radiation (IR) detectors from Hitachi Chromaster 5450 Refractive Index RI Detector (Tokyo, Japan) and Precision Instruments IOTA2 Refractive Index Detector (Marseille, France), respectively. NMR spectral data were collected using Bruker AV-500 MHz (Bruker, Rheinstetten, Germany). HR-ESI-MS was conducted on a Q Exactive™ Plus Hybrid Quadrupole Orbitrap™ Mass Spectrometer (Thermo Fisher Scientific Inc., USA).

### Extraction and Isolation

Dry materials (3.0 kg) were immersed in 95% ethanol (30 L) for seven days at room temperature. This extraction process was repeated three times in a sealed container. The liquid extract was evaporated under decreased pressure at 45 °C to yield 400 g of crude extract. The crude extract was then dispersed in water and consecutively partitioned with *n*-hexane, EtOAc, and *n*-butanol to obtain *n*-hexane, EtOAc, and *n*-BuOH fractions, respectively.

The EtOAc fraction was chromatographed on the normal phase column, effectively eluting with a gradient mobile phase of EtOAc-hexane (v/v), resulting in 10 fractions (Fr.1–10). Compound **1** (94.7 mg) was isolated from the Fr.1 fraction using semi-preparative HPLC with a normal phase, employing EtOAc-hexane (10:90, v/v) as the mobile phase. Fr.7 fraction underwent further purification through reverse-phase semi-preparative HPLC, using H_2_O–MeOH (45:55, v/v), yielding compound **2** (24.2 mg) and compound **3** (53.4 mg).

### Network pharmacology

#### Mining of phytoconstituents and targets

The phytochemicals were identified through isolation and identification processes conducted in this study, along with relevant scientific literature of *D. spathacea* (Nguyen et al. 2018a, b, 2024; Thao et al. 2021a, b). The SwissTargetPrediction, a widely recognized online platform, provides information on chemical compounds and their biological effects while predicting the primary targets of small molecules. The target genes of active compounds were predicted by using SwissTargetPrediction with the 'homo sapiens' species parameter. The target genes with a probability of ≥ 0.1 were selected as potential genes after eliminating duplicates, while compounds lacking target gene information were excluded from consideration (Que et al. 2021). Drug-likeness scores of individual active compounds were obtained from MolSoft provider (https://molsoft.com/mprop/) by submitting SMILES strings. Bioactives with positive drug-likeness scores were selected for further analysis. DrugBank (https://go.drugbank.com/) and the Human Gene Database (https://www.genecards.org/) were used to identify target genes associated with chronic bronchitis. Keyword searches, including "chronic bronchitis" and specifying the species as *Homo sapiens*, were conducted in these databases, with a disease relevance score ≥ 10. The identified target genes were combined, and redundancies were removed.

**Fig. 1:**
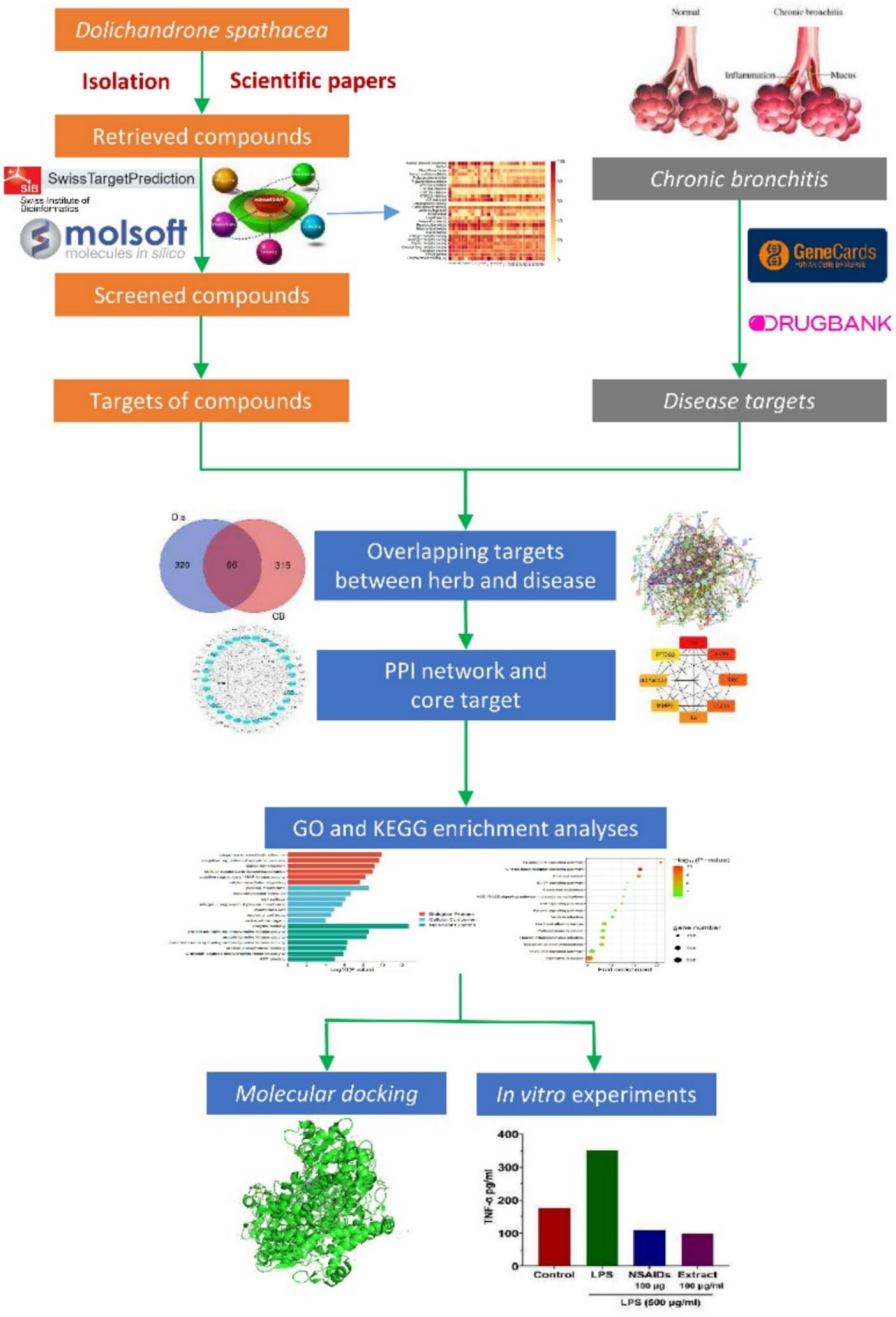
Workflow for *D. spathacea* on treating chronic bronchitis

The intersecting target genes between the bioactive compounds and the disease-associated target genes were identified and visualized using the Bioinformatics & Evolutionary Genomics platform (https://bioinformatics.psb.ugent.be/webtools/Venn/). From these analyses, bioactive compounds and botanical target genes correlated with the disease target genes, enabling the establishment of compound-target gene interactions. To illustrate these relationships, Cytoscape 3.7.2 (http://www.cytoscape.org/) was employed to construct a network diagram depicting the connections between the bioactive compounds and the disease-associated target genes (Fig. 1).

#### Prediction of pharmacokinetics and toxicokinetics of phytoconstituents

The ADMET profile of each phytoconstituent was predicted by admetSAR 2.0 (http://lmmd.ecust.edu.cn/admetsar2/). This tool was employed to estimate various parameters, including Caco-2 permeability, human intestinal absorption, P-glycoprotein (P-gp) inhibition and substrate activity, blood–brain barrier permeability, substrates for cytochrome P450 (CYP2C9, CYP3A4, CYP2D6), inhibition of cytochrome P450 (CYP2C9, CYP2C19, CYP3A4, CYP1A2, CYP2D6), ames mutagenesis, micronuclear toxicity, hepatotoxicity, acute oral toxicity, and plasma protein binding (Dwivedi et al. 2021; Thi Duong et al. 2024).

#### Gene expression and enrichment analysis

The intersecting targets were analyzed using the Search Tool for the Retrieval of Interacting Genes/Proteins (STRING, https://string-db.org/) to generate connectivity networks among them, specifying *Homo sapiens* as the gene type. The resulting connectivity network was visualized by Cytoscape 3.7.2 software. Network configuration was analyzed by evaluating node degree, which indicates the number of connections a node has with others. Nodes with degree scores exceeding twice the average were identified as main targets, highlighting their critical roles within the network.

The Database for Annotation, Visualization and Integrated Discovery (DAVID, https://david.ncifcrf.gov/), an online tool for gene classification, annotation, and pathway analysis, was employed for further analysis such as Gene Ontology (GO) and Kyoto Encyclopedia of Genes and Genomes (KEGG) pathway enrichment assessments. A significance threshold of *p* < 0.05 was applied for GO terms, and *p* < 0.01 for KEGG pathways. The top entries resulting from these analyses were visually represented. The GO enrichment assessments focus on the biological processes (BP), cellular components (CC), and molecular functions (MF) associated with the target proteins, while the KEGG enrichment assessments explore potential biological pathways and functions linked to the target genes.

### Molecular docking analysis

The experimental setup and parameter configuration closely followed the methodology previously described by Hsu et al. (2021). To delve into the binding affinity of the compounds with receptors, the CDOCKER Receptor-Ligand Interactions protocol within Discovery Studio software (DS 2021, Accelrys Software Inc., USA) was employed. The crystallographic structure of the receptors, with its PDB code obtained from the RCSB Protein Data Bank (PDB, https://www.rcsb.org), formed the basis of this study. The compounds were initially represented in 2D structures using ChemBioDraw Ultra 13.3. These 2D representations were then converted into the required 3D formats within DS 2021. Following this conversion, an energy minimization step was performed using the conjugate gradient method with a convergence criterion of 0.001 kcal/mol, and it was conducted within the CHARMm force field (Brooks et al. 1983). The resulting energy-minimized structures were subsequently subjected to molecular docking experiments. The preparation of the receptor structure (PDB code) involved the removal of water molecules and the addition of hydrogen atoms. A dedicated module was used to model the missing loop regions within the prepared protein structure. Additional steps included calculating protein ionization and protonating the protein structure, culminating in a final energy minimization phase optimized for molecular docking. The binding site was defined from PDB site records and the edit binding site module, the binding site sphere (x, y, z, radius 15) of protein was selected for molecular docking analysis. Afterward, the energy-minimized receptors-inhibited compounds were docked into the binding site of protein using the CDOCKER program embedded in DS 2021 (Hsu et al. 2021). Furthermore, for each complex, the ligand-binding free energy was calculated using the Generalized Born with Molecular Volume (GBMV) approach (Lee et al. 2003).

### In vitro experiments

#### Cell culture and viability assay

RAW 264.7 cell cultures were obtained from the American Type Culture Collection (Manassas, VA, USA), and maintained in DMEM supplemented with heat-inactivated fetal bovine serum (10%) and 1% penicillin/streptomycin at 37 °C with 5% CO_2_. The cells were incubated under these conditions during the experiment, which was performed using cells in the logarithmic growth phase.

The cytotoxicity of ethanol extract was assessed using the MTT assay. RAW264.7 macrophage cells were treated with lipopolysaccharides (Cat. No. HY-D1056, MedChemExpress, Bio-Genesis Technologies Inc., 10 µg/mL) or the ethanol extract (200 and 400 µg/mL) for 24 h in 96-well plates (2 × 10^4^ cells). After treatment, the supernatant was removed, and a medium containing MTT solution (5 mg/mL in phosphate-buffered saline) was added to each well, followed by a 2-h incubation period. The medium was discarded, and 100 µL of dimethyl sulfoxide (Duchefa Biochemie, Haarlem, The Netherlands) was added to each well to dissolve the purple formazan product, forming a colored solution. Absorbance was measured at 540 nm using a microplate reader.

#### Measurement of nitric oxide production

The production of nitric oxide (NO) was assessed by quantifying nitrite levels in the supernatants of cultured RAW 264.7 macrophage cells. The cells were seeded in 96-well culture plates at a density of 5 × 10^5^ cells/mL and pre-treated with ethanol extract at concentrations of 20, 100, 200, and 400 µg/mL, followed by stimulation with LPS (10 μg/mL) for 24 h. After the incubation period, the supernatants were collected and mixed with Griess reagent, then incubated at room temperature for 10 min. Absorbance was measured at 540 nm using a microplate reader (Molecular Devices, San Jose, CA, USA). The inhibition of NO production of the sample was calculated using the equation:

$${\text{Inhibition (\% ) = }}\left( {{1} - \, \frac{{\text{Content of NO in extract}}}{{\text{Content of NO in LPS}}}} \right) \times {100 }$$

#### Enzyme-linked immunosorbent assay (ELISA)

Aspirin is a salicylate and non-steroidal anti-inflammatory medication that has antipyretic, analgesic and anti-inflammatory effects. It is an effective treatment for certain acute or chronic inflammatory diseases. Dissolve aspirin in d.d H_2_O to prepare a stock solution with a concentration of 1 mg/mL. Seal it with paraffin and place in 4 °C refrigerator to avoid light. Dilute to the required concentration when used. RAW 264.7 cells were exposed to LPS (0.5 μg/mL) for 24 h, followed by treatment with ethanol extract (100 μg/mL) for an additional 48 h in 96-well plates, aspirin was used as a positive control. After treatment, the culture medium's supernatant was collected and centrifuged. The cellular cytokines levels in the cell culture medium were measured using mouse IL-1β and TNF-α ELISA Kit, according to the enzyme-linked immunosorbent assay manufacturer’s protocol, and the absorbance was measured at 450 nm. IL-1β ELISA Kit (Cat. No. E-EL-M0037, Elabscience, Omics Biotechnology Co., Ltd.), TNF-α ELISA Kit (Cat. No. E-EL-M3063, Elabscience, Omics Biotechnology Co., Ltd.)

### Statistical analysis

All results were reported as the average ± standard deviation (SD). Statistical evaluation was performed using one-way analysis of variance (ANOVA) to evaluate the significant differences between groups. A *p*-value of less than 0.05 was deemed statistically meaningful.

## Results

### Isolation and elucidation of phytochemicals

**Fig. 2:**
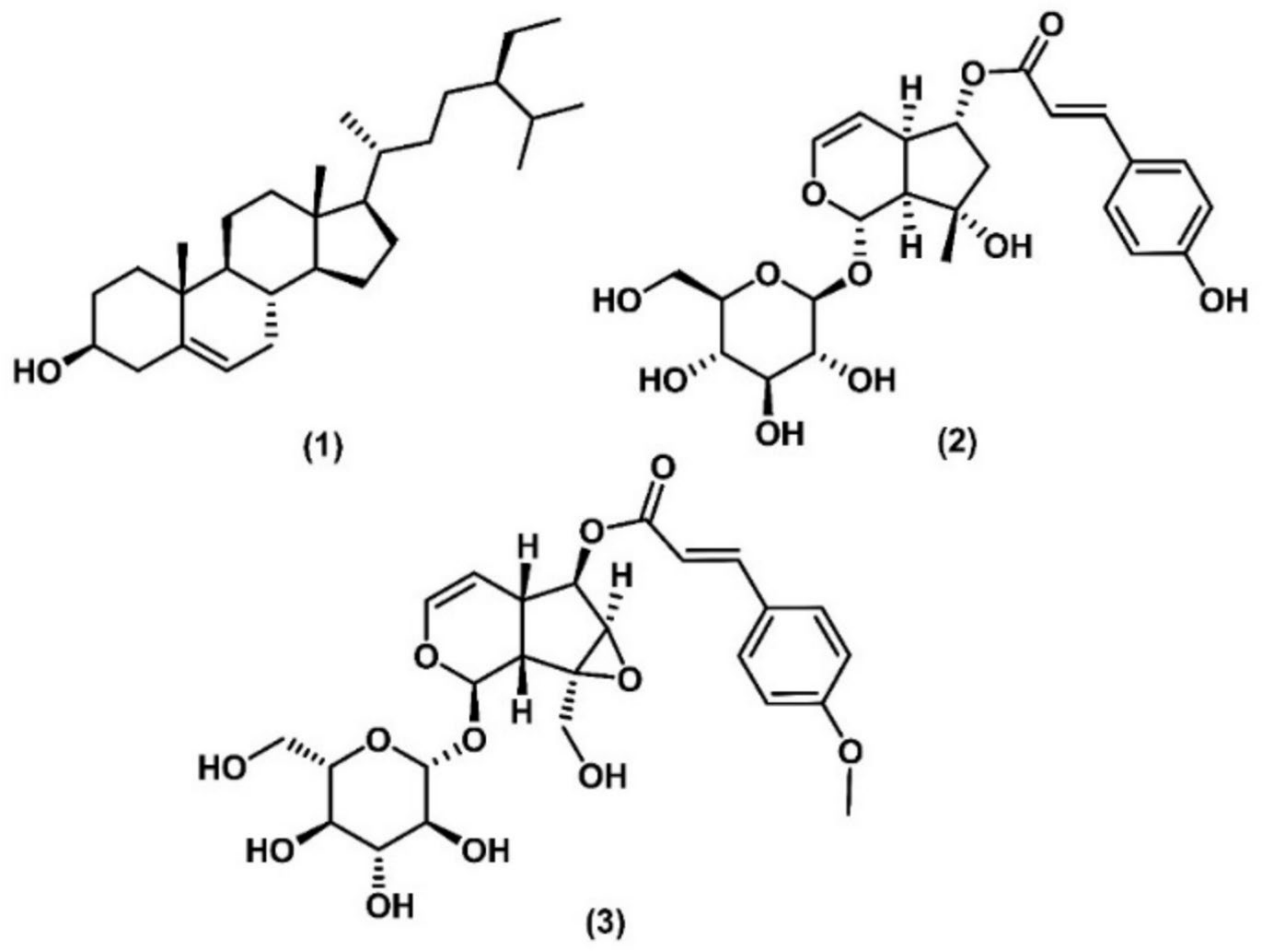
Structures of phytochemicals isolated from *D. spathacea*. (**1**), beta-sitosterol. (**2**), 6-O-*trans*-p-coumaroyl ajugol. (**3**), 6-O-trans-p-coumaroyl ajugol

The structures of all isolated compounds were elucidated using 1D and 2D NMR spectroscopy, HRESI-MS, and comparison with literature references. Three compounds were identified: beta-sitosterol (**1**) (Chang et al. 1981), 6-O-*trans*-p-coumaroyl ajugol (**2**) (Nishimura et al. 1989), and 6-O-[(*E*)-4-methoxycinnamoyl]catalpol (**3**) (Thao et al. 2021a). Among these, beta-sitosterol was isolated from this species for the first time. The structures of the three compounds of *D. spathacea* are shown in Fig. 2.

The spectral data of the compounds are provided in the Fig. S2-10 and detailed below:

Beta-sitosterol (**1**): white crystal powder, HRESI-MS [M-H_2_O + H]^+^ m/z 397.3829, C_29_H_50_O. ^1^H NMR (500 MHz, Pyr-d5) 5.45 (d, *J* = 5.1 Hz, 1H, H-6), 3.89 (tt, *J* = 10.5, 5.2 Hz, 1H, H-3), 1.09 (s, 3H, 19-CH_3_), 1.02 (d, *J* = 6.4 Hz, 3H, 21-CH_3_), 0.71 (s, 3H, 18-CH_3_). ^13^C NMR (125 MHz, Pyr-d5) δ_C_ 142.0 (C-5), 121.3 (C-6), 71.3 (C-3), 56.9 (C-14), 56.3 (C-17), 50.5 (C-9), 46.1 (C-24), 43.5 (C-4), 42.5 (C-13), 40 (C-12), 37.9 (C-1), 36.9 (C-10), 36.4 (C-20), 34.2 (C-22), 32.7 (C-2), 32.2 (C-7), 32.2 (C-8), 29.5 (C-25), 28.6 (C-16), 26.4 (C-23), 24.6 (C-15), 23.4 (C-28), 21.4 (C-11), 20 (C-27), 19.6 (C-26), 19.2 (C-19), 19.0 (C-21), 12.2 (C-29), 12.0 (C-18) (Chang et al. 1981).

6-*O*-*trans*-*p*-coumaroyl ajugol (**2**): white amorphous powder, HRESI-MS [M-H]^+^ m/z 493.1707, C_24_H_30_O_11_. ^1^H NMR (500 MHz, CD_3_OD) δ 7.62 (d, *J* = 16.0 Hz, 1H, H-γ), 7.45 (d, *J* = 8.6 Hz, 2H, H-2", 6"), 6.79 (d, *J* = 8.7 Hz, 2H, H-3", 5"), 6.34 (d, *J* = 15.9 Hz, 1H, H-β), 6.21 (dd, *J* = 6.3, 2.3 Hz, 1H, H-3), 5.49 (d, *J* = 2.5 Hz, 1H, H-1), 4.66 (d, *J* = 7.9 Hz, 1H, H-1'), 2.91 (dt, *J* = 8.9, 2.7 Hz, 1H, H-5), 2.24 (dd, *J* = 14.2, 6.5 Hz, 1H, H-7), 1.99 (dd, *J* = 14.1, 4.1 Hz, 1H, H-7), 1.39 (s, 3H, 10-CH_3_). ^13^C NMR (125 MHz, CD_3_OD) δ_C_ 169.1 (C-alpha (COO)), 161.5 (C-4"), 146.7 (C- γ), 141.1 (C-3), 131.1 (C-6"), 131.2 (C-2"), 127.1 (C-1"), 116.9 (C-3"), 116.9 (C-5"), 115.3 (C-β), 104.6 (C-4), 99.4 (C-1'), 93.4 (C-1), 80.3 (C-6), 79.2 (C-8), 78.2 (C-5'), 78 (C-3'), 74.8 (C-2'), 71.7 (C-4'), 62.9 (C-6'), 51.6 (C-9), 47.9 (C-7), 39.4 (C-5), 26 (C-10) (Nishimura et al. 1989).

6-*O*-[(*E*)-4-methoxycinnamoyl]catalpol (**3**): white amorphous powder, HRESI-MS [M + FA-H]^−^ m/z 567.1724, C_25_H_30_O_12_. ^1^H NMR (500 MHz, CD_3_OD) δ 7.72 (d, *J* = 15.9 Hz, 1H, H-7"), 7.59 (d, *J* = 8.9 Hz, 2H, H-2", 6"), 6.98 (d, *J* = 8.8 Hz, 2H, H-3", 5"), 6.45 (d, *J* = 16.0 Hz, 1H, H-8"), 6.39 (dd, *J* = 5.9, 1.7 Hz, 1H, H-3), 5.18 (d, *J* = 9.2 Hz, 1H, H-1), 5.05 (dd, *J* = 7.8, 1.3 Hz, 1H, H-6), 5.00 (dd, *J* = 6.0, 4.1 Hz, 1H, H-4), 4.81 (d, *J* = 7.9 Hz, 1H, H-1'), 4.18 (d, *J* = 13.1 Hz, 1H, H-10), 3.94 (dd, *J* = 12.0, 2.2 Hz, 1H, H-6'), 3.85 (s, 3H, 4"-OCH_3_), 3.83 (d, *J* = 1.5 Hz, 1H, H-7), 3.66 (dd, *J* = 12.0, 6.7 Hz, 1H, H-6'). ^13^C NMR (125 MHz, CD_3_OD) δ_C_ 168.6 (C-9"), 163.1 (C-4"), 146.7 (C-7"), 142.2 (C-3), 130.9 (C-6"), 130.9 (C-2"), 128.1 (C-1"), 115.3 (C-8"), 115.3 (C-3",5"), 102.7 (C-4), 99.5 (C-1'), 94.9 (C-1), 81.2 (C-6), 78.5 (C-5'), 77.5 (C-3'), 74.7 (C-2'), 71.6 (C-4'), 66.6 (C-8), 62.7 (C-6'), 61.1 (C-10), 60.1 (C-7), 55.7 (C-4"), 43 (C-9), 36.6 (C-5) (Thao et al. 2021a).

### Network pharmacology analysis

#### Mining of phytoconstituents and targets

The phytochemicals of *D. spathacea* were obtained from the isolation and scientific papers, resulting in 59 compounds. After predicting target genes using SwissTargetPrediction with the ‘*homo sapiens*’ species setting, targets of phytochemicals with a probability ≥ 0.1 were considered, resulting in 49 compounds. Chronic bronchitis target genes (1,410 genes) were obtained from GeneCards. Among these, 323 prospective target genes, which consisted of genes with relevance scores ≥ 10, were identified. Additionally, 104 genes associated with disease target genes were identified from 18 drugs used to treat chronic bronchitis, sourced from DrugBank (https://www.drugbank.ca/). After combining the data and removing duplicates, a total of 381 genes were collected.

**Fig. 3:**
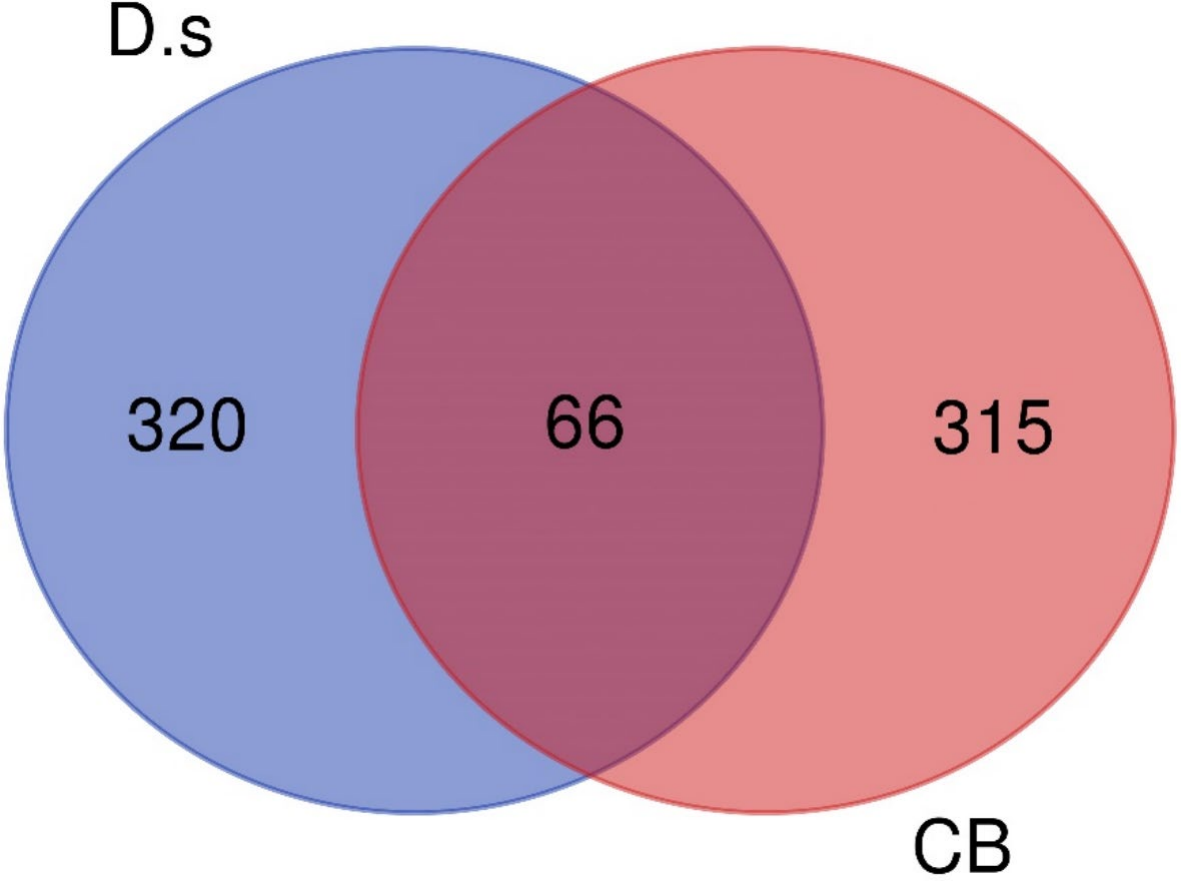
Venn diagram of the overlapping targets in *D. spathacea* and chronic bronchitis

After identifying the 381 genes associated with chronic bronchitis from Drugbank and Genecards, 42 compounds were found to be linked to these genes. Thirty phytochemicals had drug-likeness scores above 0, with salvionoside B scoring the highest at 0.79 (Table 1). By overlapping the 386 target genes of *D. spathacea* with 381 target genes associated with CB, a total of 66 intersecting target genes were identified, as depicted in the Venn diagram (Fig. 3). The details of overlapping target genes are shown in Table S1.

**Table 1:** Drug-likeness score and number of genes modulated by phytoconstituents

No.	Phytoconstituents	Number of HBA	Number of HBD	MolLogP	MolLogS		DLS	Gene count
					Log (mol L^−1^)	Mg L^−1^		
1	Luteolin	6	4	2.78	− 3.11	224.49	0.38	21
2	Dolichandrone B	3	2	6.06	− 5.46	1.64	0.22	17
3	Salvionoside B	11	6	− 0.35	− 0.52	152,263.05	0.79	16
4	Uncaric acid	5	4	5.14	− 4.35	21.95	0.58	16
5	Oleanolic acid	3	2	6.48	− 5.58	1.2	0.37	14
6	Ursolic acid	3	2	6.46	− 5.6	1.15	0.66	13
7	*6S,9S* roseoside	8	5	− 0.13	− 0.58	100,591.8	0.33	9
8	6-*O-E*-caffeoyl-ajugol	12	7	− 0.19	− 1.51	15,860.54	0.53	8
9	Pomolic acid	4	3	5.91	− 5.2	3	0.32	8
10	Beta-sitosterol	1	1	8.45	− 6.34	0.19	0.78	7
11	Luteolin-7-*O*-β-D-glucopyranoside	11	7	0.47	− 1.66	9897.27	0.60	7
12	6-*O*-p-E-coumaroyl-ajugol	11	6	0.2	− 1.62	11,843.34	0.12	7
13	luteolin-7-*O*-β-D-glucuronide	12	7	0.84	− 1.79	7516.87	0.71	6
14	5α-stigmastane-3,6-dione	2	0	7	− 5.82	0.65	0.65	6
15	decaffeoylacteoside	12	8	− 2.33	− 1.16	31,777.9	0.31	5
16	1-(α-L-rhamnosy l(1–6)-β-D-glucopyranosyloxy)-3,4,5-trimethoxybenzene	13	6	2.3	− 0.69	101,331.56	0.15	5
17	Luteolin-7-*O*-rutinoside	16	10	− 1.4	− 1.56	16,841.82	0.61	3
18	Cistanoside F	13	8	− 1.26	− 1.43	18,309.06	0.38	3
19	(-)-isolariciresinol-3α-*O*-β-D-glucopyranoside	11	7	− 0.07	− 1.33	24,146.56	0.78	2
20	verminoside	13	7	− 1	− 1.42	20,045.09	0.28	2
21	minecoside	13	6	− 0.49	− 1.45	19,246.35	0.06	2
22	6’’*R-O*-(2*E*)-8-hydroxy-2,6-dimethyl-2-octenoyl-ajugol	11	6	0.78	− 1.04	46,902.5	0.68	1
23	Verbascoside	15	9	− 0.15	− 1.44	22,890.62	0.51	1
24	Nemorososide	11	6	0.58	− 1.02	49,509.33	0.5	1
25	Martynoside	15	7	0.69	− 1.55	18,534.69	0.44	1
26	Isoverbascoside	15	9	− 0.2	− 1.41	24,502.34	0.42	1
27	6’’*R-O*-(2*E*)-8-hydroxy-2,6-dimethyl-2-octenoyl)-catalpol	12	6	0.05	− 0.62	128,019.9	0.41	1
28	6-*O-E*-isoferuloylajugol	12	6	0.31	− 1.55	14,808.4	0.29	1
29	Nemoroside	12	6	− 0.15	− 0.46	183,990.92	0.21	1
30	6’’(*Z*)-nemoroside	12	6	− 0.15	− 0.46	183,990.92	0.21	1

HBA, hydrogen bond acceptor; HBD, hydrogen bond donor; MolLogP, molecular logarithm of partition coefficient; MolLogS, molecular logarithm of solubility; DLS, drug-likeness score

**Fig. 4:**
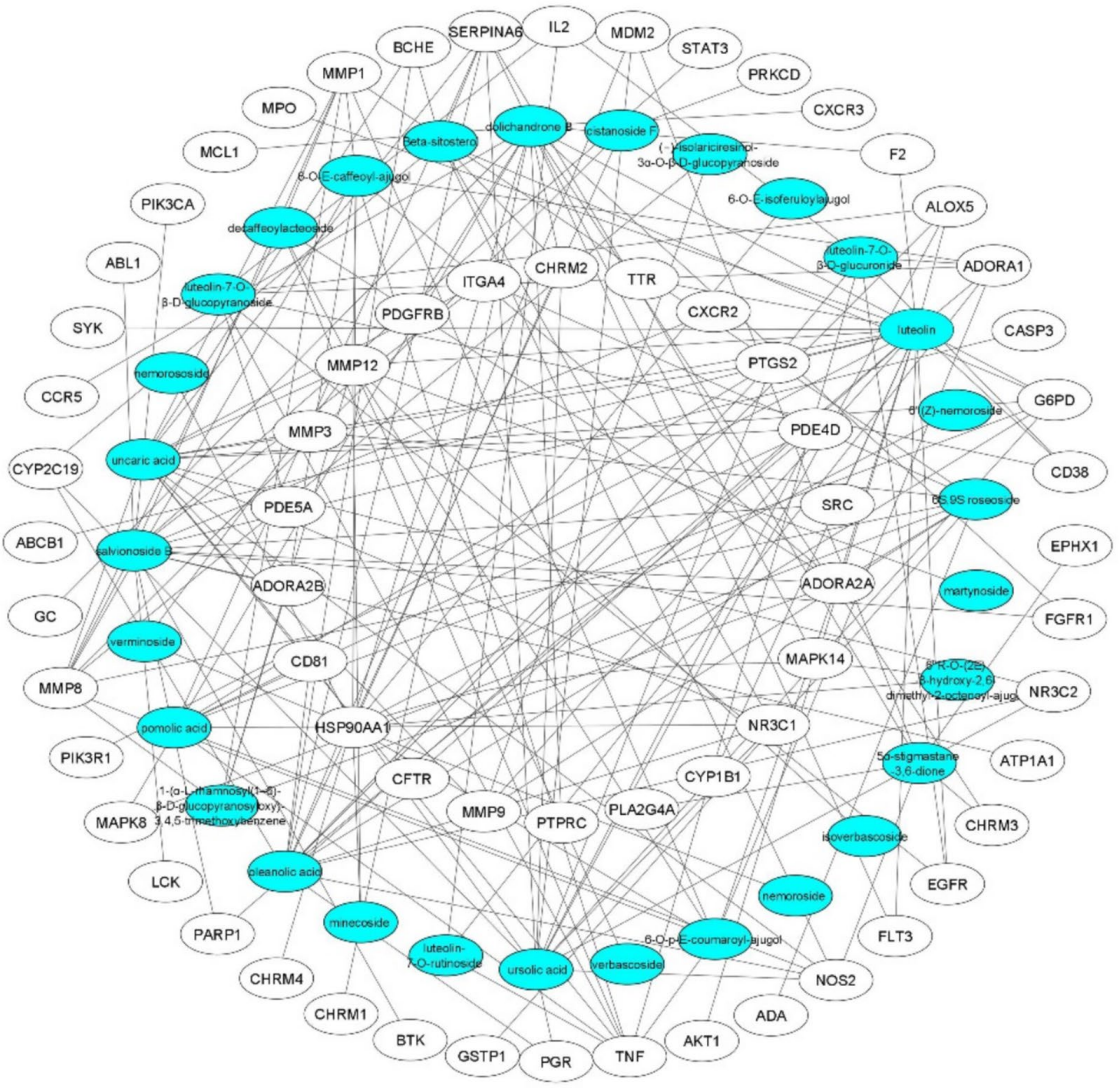
The network of target genes and related bioactives. The elliptical white nodes represent the target genes, and the elliptical blue nodes represent the bioactive compounds

Cytoscape 3.7.2 software was used to establish the drug-target interaction network, providing further insights into the mechanism of *D. spathacea* in treating chronic bronchitis (Fig. 4). This network visually represents the relationships between the bioactive compounds and the target genes involved in CB.

#### ADMET profile of bioactives

**Fig. 5:**
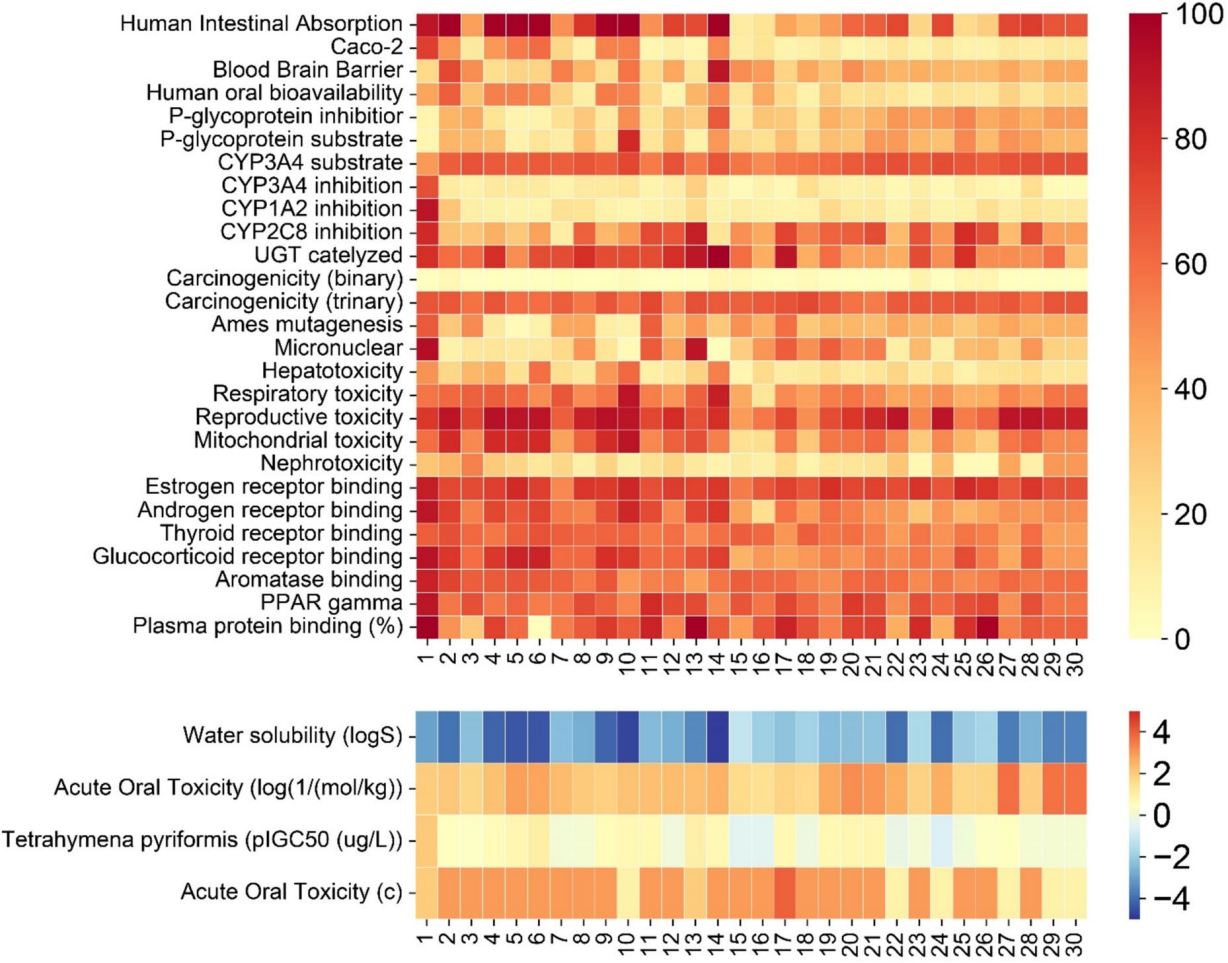
The ADMET profile of bioactive compounds of *D. spathacea* via heatmap. Encoding as follows: (1) luteolin, (2) dolichandrone B, (3) salvionoside B, (4) uncaric acid, (5) oleanolic acid, (6) ursolic acid, (7) *6S,9S* roseoside, (8) 6-*O-E*-caffeoyl-ajugol, (9) pomolic acid, (10) beta-sitosterol, (11) luteolin-7-*O-*β-D-glucopyranoside, (12) 6-*O-p-E*-coumaroyl-ajugol, (13) luteolin-7-*O-*β-D-glucuronide, (14) 5α-stigmastane-3,6-dione, (15) decaffeoylacteoside, (16) 1-(α-L-rhamnosy l(1–6)-β-D-glucopyranosyloxy)-3,4,5-trimethoxybenzene, (17) luteolin-7-*O*-rutinoside, (18) cistanoside F, (19) (-)-isolariciresinol-3α-*O*-β-D-glucopyranoside, (20) verminoside, (21) minecoside, (22) 6’’*R-O*-(2*E*)-8-hydroxy-2,6-dimethyl-2-octenoyl-ajugol, (23) verbascoside, (24) nemorososide, (25) martynoside, (26) isoverbascoside, (27) 6’’*R-O*-(2*E*)-8-hydroxy-2,6-dimethyl-2-octenoyl)-catalpol, (28) 6-*O-E*-isoferuloylajugol, (29) nemoroside, and (30) 6’’(*Z*)-nemorosid

The pharmacokinetics and toxicokinetics of each bioactive are illustrated in a heatmap showing Caco-2 permeability (Caco-2p), human intestinal absorption (HIA), blood–brain barrier (BBB) permeability, CYP3A4, CYP2C8, CYP1A2 inhibitor/substrate, P-glycoprotein (p-gp) inhibitor/ substrate, ames mutagenesis, hepatotoxicity, watersolubility, acute oral toxicity, and plasma protein binding. The ADMET profiles of bioactive compounds were illustrated as a heatmap (Fig. 5). Dolichandrone B, uncaric acid, oleanolic acid, ursolic acid, pomolic acid, beta-sitosterol, and 5α-stigmastane-3,6-dione were predicted to have good intestinal absorption (probability > 85%). All compounds from *D. spathacea* were metabolized through the CYP3A4 enzyme and inhibited CYP2C8 enzyme. Almost all compounds potentially bind to plasma proteins, except for ursolic acid.

#### Enrichment and network analysis

**Fig. 6:**
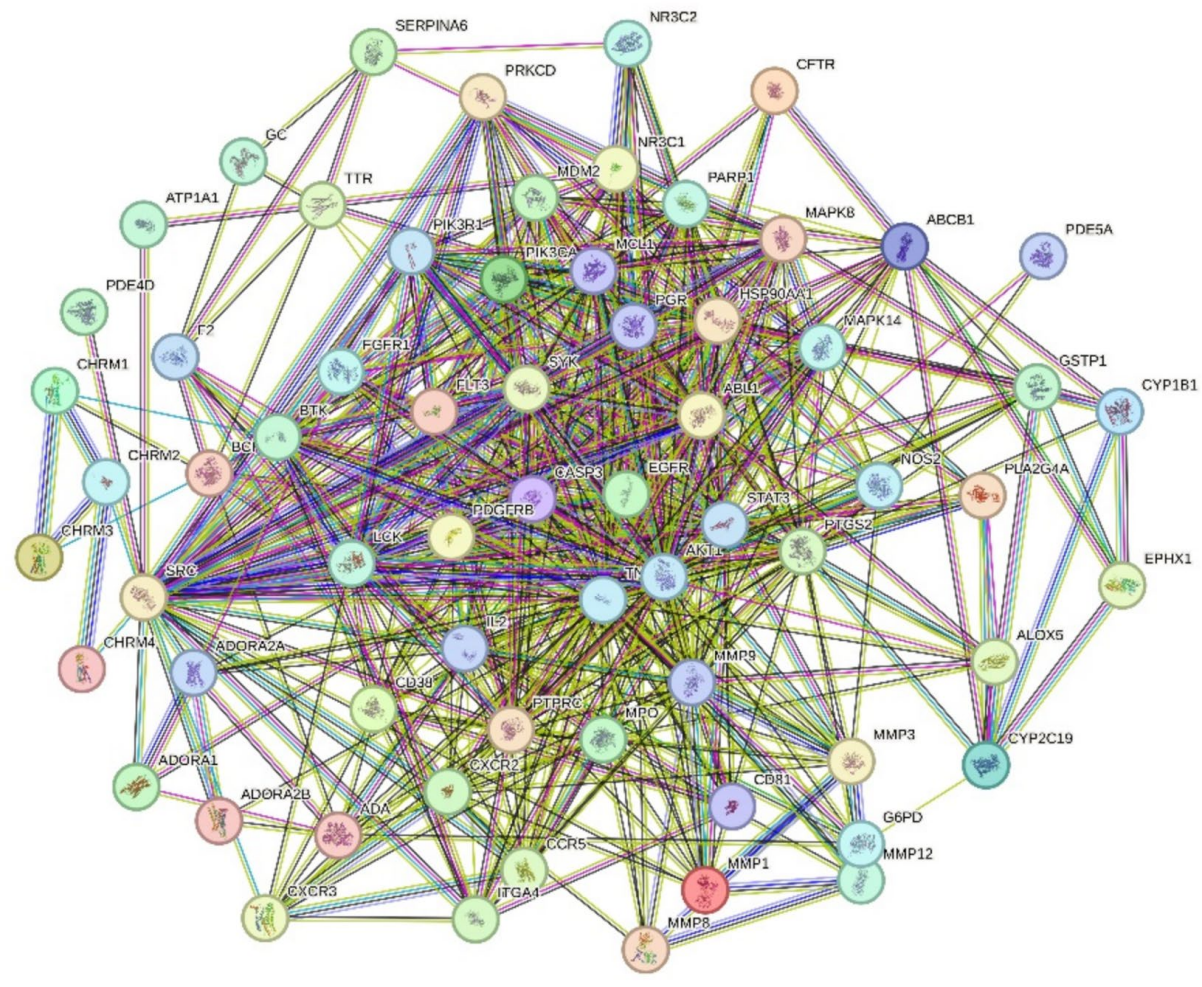
PPI network of 66 gene targets of *D. spathacea* against CB. Edges represent protein–protein associations

The protein–protein interaction (PPI) network derived from the 66 overlapping targets consists of 66 nodes and 559 edges. The average node degree is 16.9, and the p-value for PPI enrichment is less than 1.0e−16, as illustrated in Fig. 6.

**Fig. 7:**
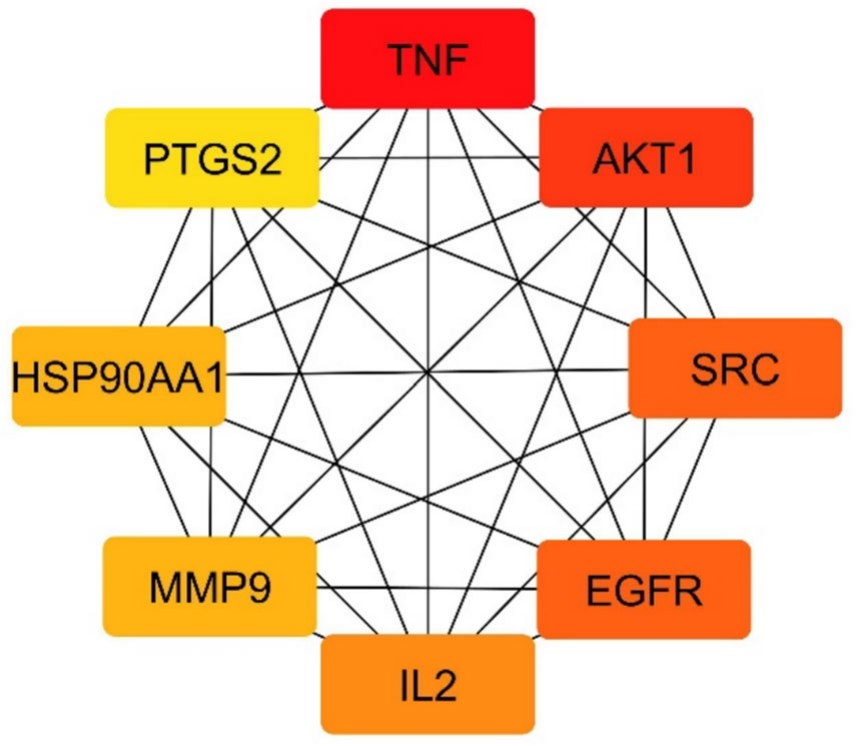
The PPI network of 8 main target genes of *D. spathacea* in treating CB. Screened by degree value, darker nodes indicate higher values

A topological analysis identified 8 main target genes based on a twofold median degree value. These core target genes include TNF, AKT1, SRC, EGFR, IL2, MMP-9, HSP90AA1, and PTGS2. A network diagram was constructed to visualize the interactions among these 8 core target genes, as shown in Fig. 7. Each target gene is represented by a circular node, with nodes shaded from dark red to light red. Darker nodes indicate higher degree values, signifying the importance of the corresponding target genes. Among them, TNF had the highest degree value (degree = 49), followed by AKT1 (degree = 42), SRC, and EGFR (degree = 39), IL (degree = 37), MMP-9, and HSP90AA1 (degree = 35), and PTGS2 (degree = 34).

**Fig. 8:**
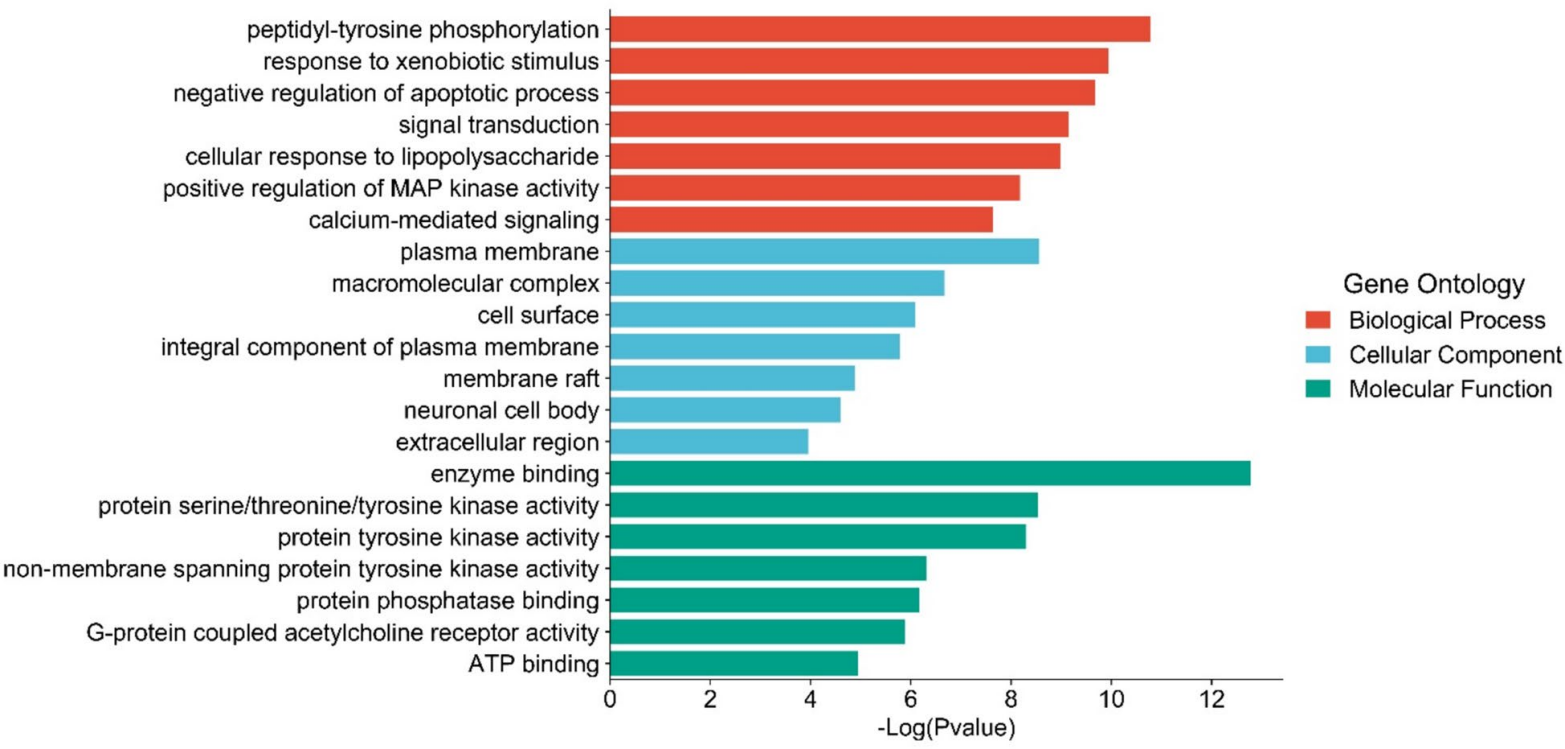
The top 7 results of the GO enrichment analysis for the 66 overlapping target genes

The Gene Ontology enrichment assessment was conducted on the 66 overlapping targets, resulting in 351 significant GO terms (*p* < 0.05). These terms encompassed 259 entries related to biological processes (BP), 44 entries related to cellular components (CC), and 48 entries related to molecular functions (MF). Among the top 7 biological process terms, the following processes were highly enriched: peptidyl-tyrosine phosphorylation (GO:0018108), response to xenobiotic stimulus (GO:0009410), negative regulation of apoptotic process (GO:0043066), signal transduction (GO:0007165), cellular response to lipopolysaccharide (GO:0071222), positive regulation of MAP kinase activity (GO:0043406), and calcium-mediated signaling (GO:0019722). These biological processes were closely associated with specific molecular functions, including enzyme binding (GO:0019899), protein serine/threonine/tyrosine kinase activity (GO:0004712), protein tyrosine kinase activity (GO:0004713), non-membrane spanning protein tyrosine kinase activity (GO:0004715), protein phosphatase binding (GO:0019903), G-protein coupled acetylcholine receptor activity (GO:0016907), and ATP binding (GO:0005524). Furthermore, these processes primarily occurred in specific cellular locations, such as plasma membrane (GO:0005886), macromolecular complex (GO:0032991), cell surface (GO:0009986), an integral component of the plasma membrane (GO:0005887), membrane raft (GO:0045121), neuronal cell body (GO:0043025), and extracellular region (GO:0005576). The top 7 GO terms are presented in Fig. 8.

**Fig. 9:**
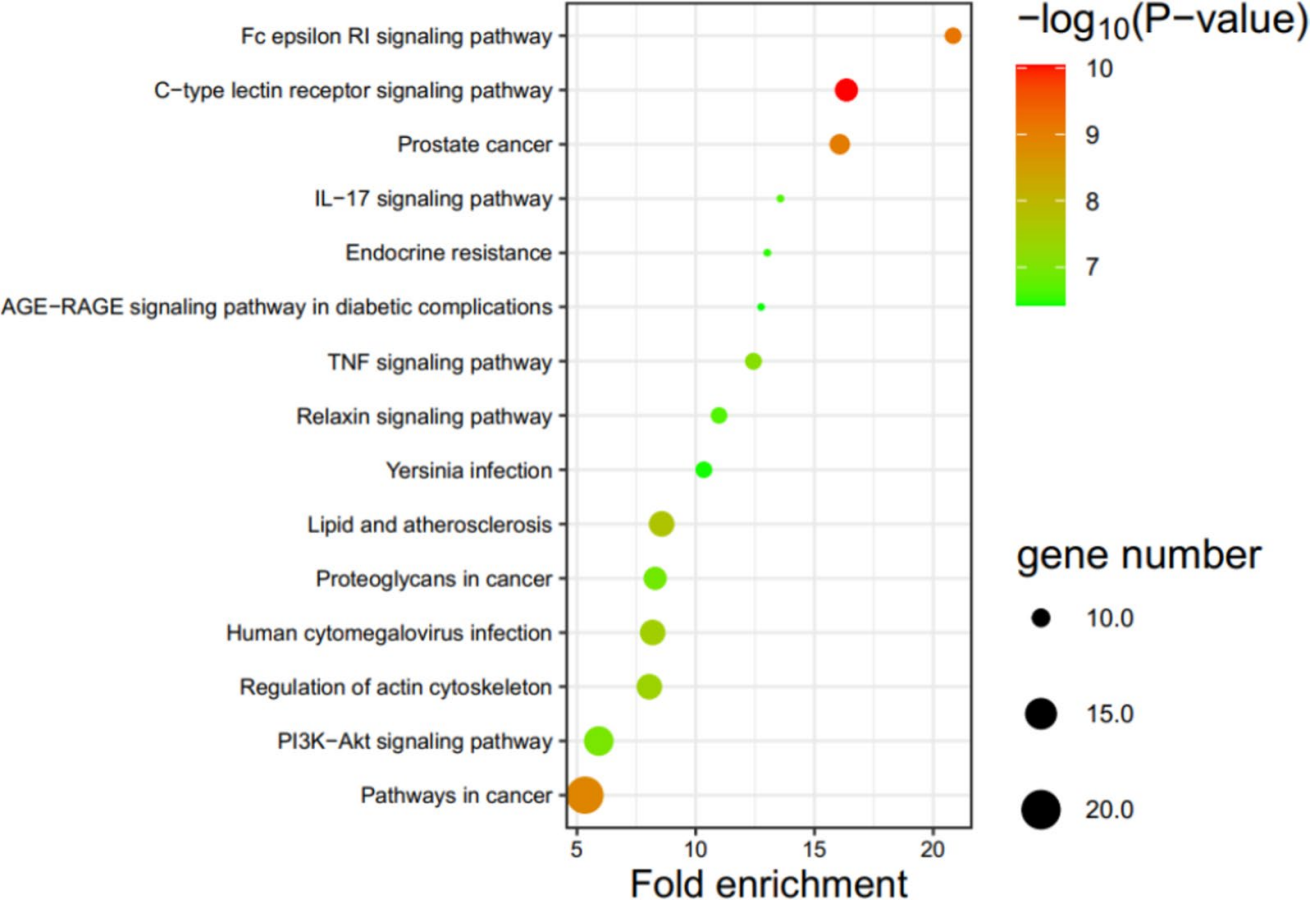
The KEGG pathway analysis of potential target genes. The color of each term represents the different p-values and the size of each term reflects the gene counts

The KEGG pathway enrichment analysis revealed that the 66 overlapping target genes were significantly enriched in 107 pathways (*p* < 0.01). To visualize the results, bubble diagrams were created, selecting the top 15 pathways (Fig. 9). The main KEGG pathways were prominently involved in chronic bronchitis, including the TNF signaling pathway (hsa04668), and IL-17 signaling pathway (hsa04657). The bubble sizes represent the number of the genes enriched in each pathway, while the intensity of colors indicates the relevance of the enrichment, with greener colors signifying smaller-log (*p*-value) and more significant results.

To illustrate the association of the regulatory target genes of this species with these pathways, three separate diagrams were constructed, showcasing the relationship between the target genes and TNF, and IL-17 signaling pathways (Figs. S11 and S12, respectively).

**Fig. 10:**
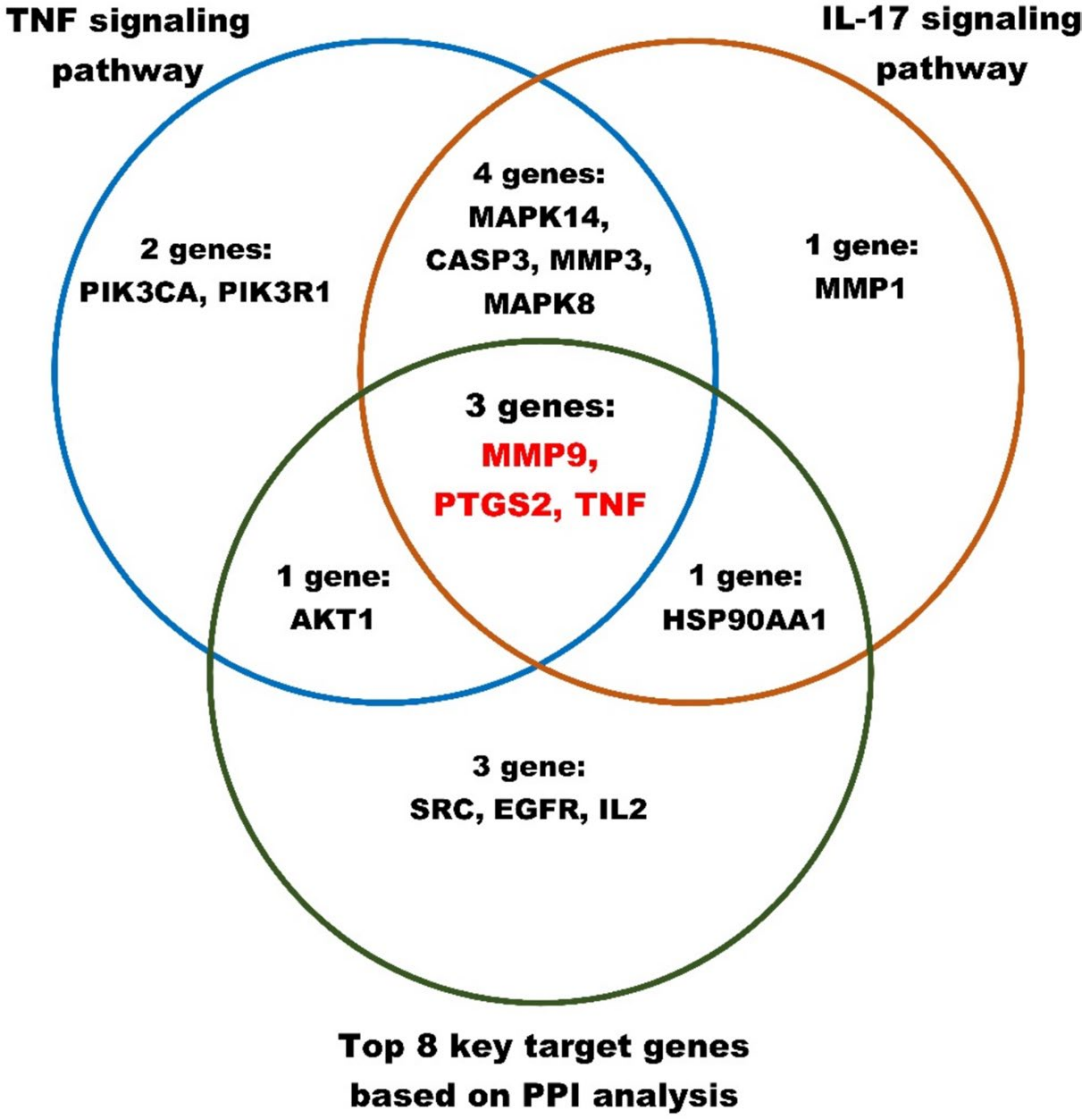
Overlapping main target genes based on PPI analysis and genes belonging to TNF, IL-17 signaling pathways

The intersection between the TOP 8 target genes based on PPI analysis and the genes involved in the TNF and IL-17 signaling pathways is presented in Fig. 10.

After network analysis of herb-compounds-hub target genes, two pathways, the nodes of main target genes including MMP-9, PTGS2, and TNF (Fig. 10). These main target genes were considered the key target genes. Furthermore, six key bioactive compounds of *D. spathacea*, namely luteolin, dolichandrone B, salvionoside B, uncaric acid, oleanolic acid, and ursolic acid were identified.

### Molecular docking analysis

The binding affinity of selected bioactive compounds with key target proteins, TNFR1 (PDB: 2AZ5), COX-2 (PDB: 5F1A), and MMP-9 (PDB: 6ESM), was evaluated using the Cdocker algorithm in Discovery Studio software. The docking results, expressed as docking affinity values (kcal/mol), are summarized in Table 2. The lower docking affinity values represent stronger binding interactions between the ligand and the target protein.

**Table 2:** Docking affinity values of the key bioactive compounds with target proteins

Key bioactive compounds	Docking affinity with target proteins (kcal/mol)		
	TNFR1 (PDB: 2AZ5)	COX-2 (PDB: 5F1A)	MMP-9 (PDB: 6ESM)
Luteolin	− 34.2596	− 40.9639	− 11.6525
Dolichandrone B	− 55.6898	− 65.4134	− 0.2125
Salvionoside B	− 43.5328	− 66.5444	14.3086
Uncaric acid	− 34.5054	− 25.2255	16.6553
Oleanolic acid	− 58.8954	− 57.0037	21.3067
Ursolic acid	− 25.1417	− 36.0138	− 4.9062
*Aspirin	− 26.4970	− 30.6013	− 18.7564

*Reference drug

Among the compounds, oleanolic acid and salvionoside B demonstrated the highest binding affinity with TNFR1 (− 58.8954 kcal/mol) and COX-2 (− 66.5444 kcal/mol), respectively. In addition, luteolin exhibited the strongest interaction with MMP-9 (− 11.6525 kcal/mol). Luteolin and dolichandrone B showed notable binding affinities across all targets, with dolichandrone B significantly outperforming the reference compound aspirin (TNFR1, − 26.4970 kcal/mol and COX-2, − 30.6013 kcal/mol).

**Fig. 11:**
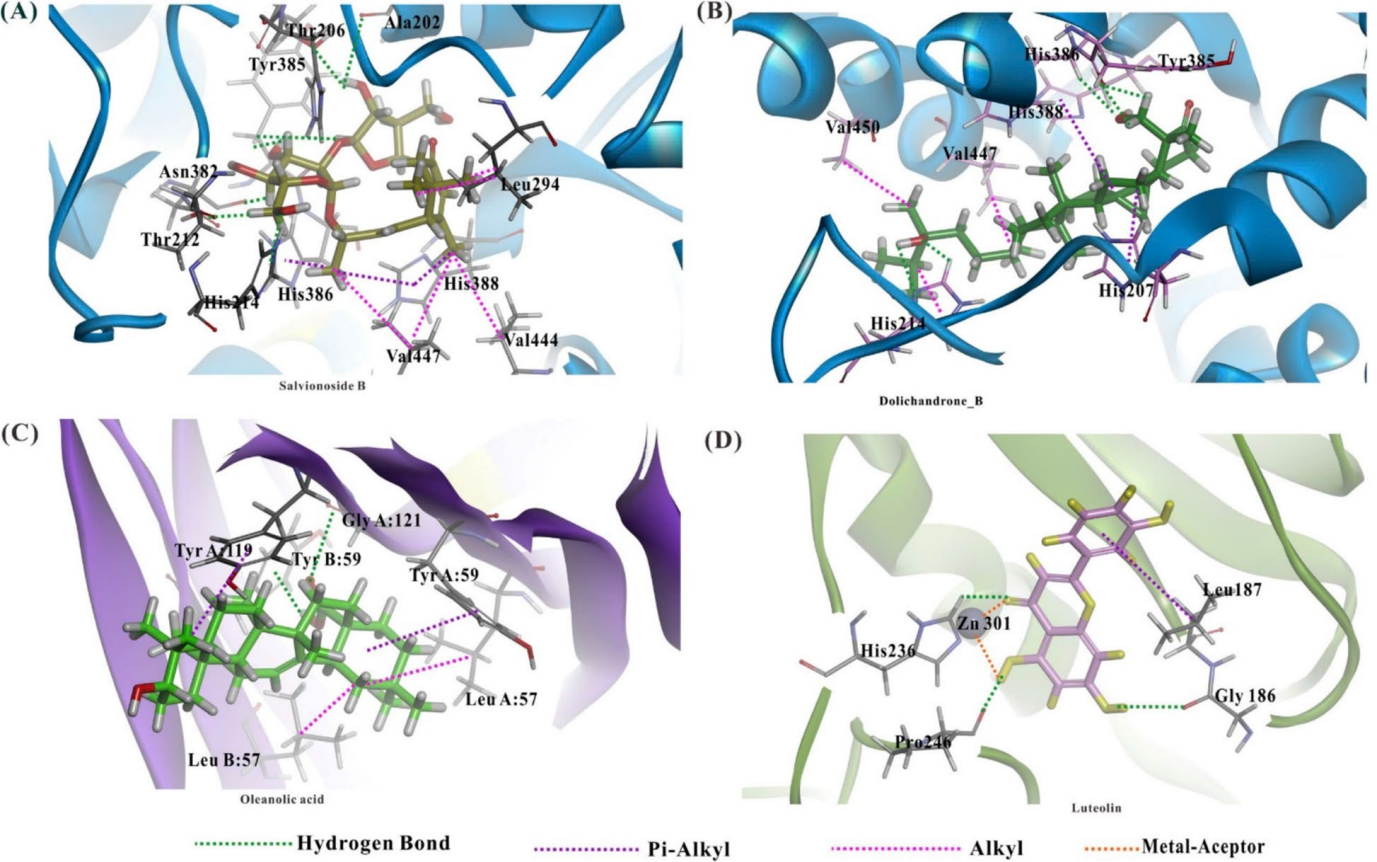
Computational docking of key target proteins and key bioactive compounds. **A, B** salvionoside B and dolichandrone B interacted with COX-2 (PDB: 5F1A), respectively. **C** oleanolic acid interacted with TNFR1 (PDB: 2AZ5). **D** luteolin interacted with MMP-9 (PDB: 6ESM)

The docking results revealed strong binding interactions between salvionoside B, dolichandrone B and COX-2; oleanolic acid and TNFR1; luteolin and MMP-9. Salvionoside B (Fig. 11A) formed multiple hydrogen bonds with active site residues, including Ala202, Thr206, His207, Thr212, His214, Thr292, Asn382, and Tyr385. Additionally, π-alkyl and alkyl interactions were observed with His386, His388 and Leu294, Val444, Val447, respectively, stabilizing the compound within the COX-2 binding pocket. Dolichandrone B (Fig. 11B) exhibited similar interactions, engaging in hydrogen bonding with His214, His386, Tyr385 and forming alkyl and π-alkyl interactions with His207, His388, Ala447, and Ala450. Oleanolic acid (Fig. 11C) demonstrated a strong affinity for TNFR1, engaging in multiple hydrogen bonds with TyrB59 and GlyA121. Pi-alkyl and alkyl interactions with TyrA59, TyrA119 and LeuA57, LeuB57. Luteolin (Fig. 11D) exhibited strong binding affinity towards MMP-9, forming hydrogen bonds with Gly186, His236 and Pro246. A crucial metal-acceptor interaction with Zn301 was observed, which is essential for MMP-9 inhibition. Additionally, pi-alkyl interaction with Leu187 further contributed to the ligand's stability within the binding pocket.

### In vitro experiments

#### Inhibitory effect of ethanol extract on NO production

The influence of ethanol extract on inflammation was investigated by measuring the levels of NO secreted upon LPS induction in cell culture. Ethanol extract with four doses (20, 100, 200, and 400 μg/mL) was applied to the cells before LPS induction, and NO production was evaluated. Dexamethasone, widely used in anti-inflammatory therapy, served as a positive control at concentrations of 0.8, 4, 20, and 100 μM. Ethanol extract strongly inhibited NO production in a dose-dependent manner. Notably, at 100 and 200 μg/mL, the ethanol extract inhibited NO production by more than 86%, and 91%, respectively, compared to untreated samples. The IC_50_ value was determined to be 25.34 μg/mL, shown in Table S2. These results indicate that ethanol extract efficiently inhibits nitrite release from LPS-induced RAW 264.7 macrophage cells.

#### The ethanol extract represses pro-inflammatory cytokine expression in RAW 264.7 Cells

**Fig. 12:**
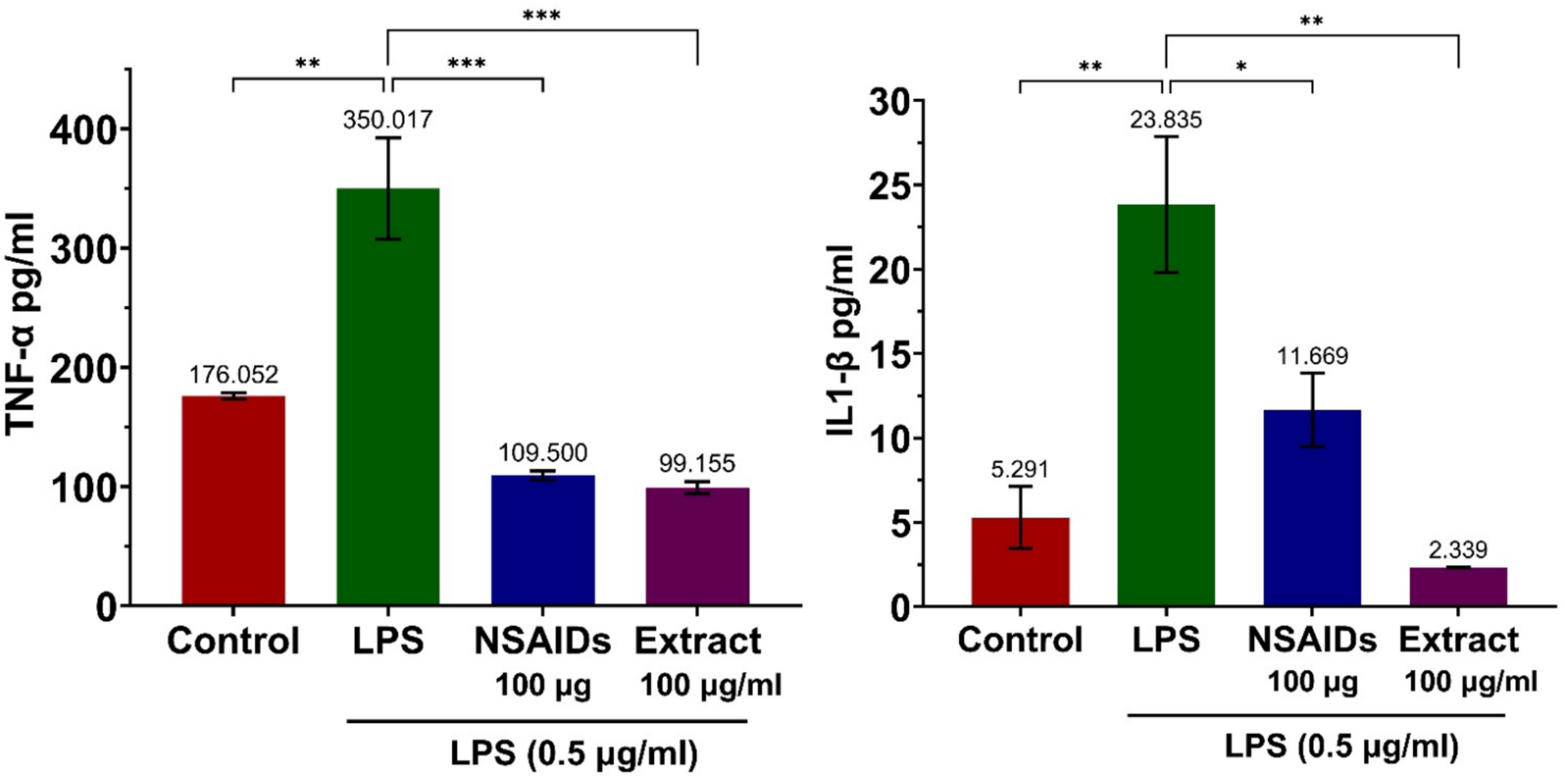
Effect of ethanol extract on LPS-induced cytokine production. **A** TNF-α, **B** IL-1β. p-values were derived from one-way ANOVA with Dunnett’s multiple comparison tests. *p-value < 0.05, **p-value < 0.01, ***p-value < 0.001. The error bars represent the standard deviation (SD) of the mean

The effect of ethanol extract on TNF-α and IL-1β production induced by LPS was investigated using ELISA analysis. Cells pretreated with ethanol extract at various concentrations were stimulated with LPS, and the levels of TNF-α and IL-1β were assessed. As shown in Fig. 12A, the ethanol extract effectively inhibited TNF-α production, achieving a 71.67% reduction at 100 μg/mL. Similarly, the ethanol extract suppressed IL-1β production, achieving a 90.22% reduction, as presented in Fig. 12B. The NSAIDs (aspirin) were used as a positive control to compare the anti-inflammatory effects of the ethanol extract. The results indicate that aspirin (100 μg/mL) significantly reduced TNF-α and IL-1β levels in LPS-stimulated cells. Specifically, TNF-α levels in the NSAID-treated group were 109.500 pg/mL, which was comparable to 99.155 pg/mL in the ethanol extract-treated group. Similarly, IL-1β levels decreased from 23.835 pg/mL in the LPS group to 11.669 pg/mL in the NSAID-treated group, as shown in Fig. 12.

## Discussion

Network pharmacology is widely used to uncover the biological mechanisms underlying traditional Chinese medicine formulas. This approach involves constructing complex interaction networks that incorporate bioactives, their targets and associated various biological functions (Luo et al., 2020). In this study, three phytochemicals (beta-sitosterol, 6-O-*trans-*p-coumaroyl ajugol, 6-O-[(*E*)-4-methoxycinnamoyl]catalpol) were isolated from *D. spathacea* along with 56 other compounds identified from the literature references.

Although previous have reported the anti-inflammatory properties of this species, the precise mechanism underlying these effects remains unclear. Network pharmacology provides a promising approach to investigating the fundamental pharmacological mechanisms of *D. spathacea*. In this study, network pharmacological analysis identified six key bioactive compounds, three key target genes, and two major signaling pathways responsible for their therapeutic effects*.*

Based on the network assessment of correlations between phytochemicals and disease-target genes—luteolin, dolichandrone B, salvionoside B, uncaric acid, oleanolic acid, and ursolic acid—were identified as key bioactive compounds, regulating at least eight key target genes. Four of these bioactives are associated with steroids, which exhibit bioactivities such as anti-inflammatory, antiviral, and antimicrobial effects. Luteolin, a flavonoid, demonstrates a diverse range of biological activities, including anti-inflammatory, antioxidant, antimicrobial, and anticancer properties (López-Lázaro 2009). Salvionoside B, an aliphatic alcohol glycoside, has been previously reported for its anti-inflammatory properties (Huang et al. 2022).

Luteolin exerts anti-inflammatory effects by inhibiting inflammatory factors such as TNF-α, COX-2, IL-1β, and IL-6, as well as by regulating signaling pathways like NF-κB and JAK-STAT (Gendrisch et al. 2021). It suppresses NF-κB pathway activation in vivo by downregulating miR-132 and inhibits PM2.5-stimulated pro-inflammatory COX-2 and iNOS in MH-S cells (Hsieh et al. 2022; Liu and Meng 2018). Ursolic acid, a pentacyclic triterpene, is recognized for its potent anti-inflammatory and antioxidant effects. It reduces levels of inflammatory cytokines such as TNF-α, IL-1β, and IL-6 produced by immune cells while modulating reactive oxygen species (ROS) and nitric oxide (NO) formation. Studies have shown that ursolic acid significantly lowers serum levels of TNF-α, IL-6, and IL-1β, and inhibits the expression of inducible nitric oxide synthase (iNOS) and cyclooxygenase-2 (COX-2) in the lungs, reducing nitric oxide and prostaglandin E2 production. As an effective COX-2 inhibitor, it can suppress inflammation progression (Zhang et al. 2022). Oleanolic acid has been shown to reduce airway inflammation and Th2-mediated allergic asthma induced by ovalbumin in asthmatic mice. It modulates transcription factors T-bet and GATA-3 (Kim et al. 2014). Oleanolic acid exhibits strong anti-inflammatory effects and is effective against various types of inflammation, including enteritis and vasculitis (Chai et al. 2015). Additionally, in vitro studies revealed that certain derivatives of oleanolic acid possess significant anti-inflammatory activities (Yan et al. 2019). Dolichandrone B demonstrated high cytotoxic activity against the KB cell line, while salvionoside B exhibited strong anti-inflammatory effects (Nguyen et al. 2018b).

Through the establishment of a PPI network of overlapping target genes between phytochemicals and chronic bronchitis, 8 main target genes were identified: TNF, AKT1, SRC, EGFR, IL2, MMP-9, HSP90AA1, and PTGS2 (COX-2). Tumor necrosis factor (TNF) is a cytokine with well-known proinflammatory properties that contribute to various diseases. TRAF2 (TNF-α), a TNF receptor-associated factor, has been identified as a key mediator in TNF signaling. TNF-α is the primary cytokine within the TNF pathway; its levels are elevated in COPD and it triggers the release of additional pro-inflammatory factors. The activation of NF-κB and MAPKs, essential components of the TNF signaling pathway, is crucial for inducing numerous cytokines and immune-regulatory proteins, playing a significant role in inflammatory responses (Liu 2021, 2005). COX-2 (PTGS2 gene) is an enzyme produced in response to various stimuli and is associated with inflammation and tumor cell proliferation. While minimally expressed in normal cells, COX-2 is highly expressed in rapidly dividing cells. It can activate macrophages and other inflammatory cells, transforming the inflammatory site into an early-stage cancer microenvironment, thereby exhibiting carcinogenic effects (Alexanian & Sorokin 2017; Sun et al. 2020). Akt, a serine/threonine protein kinase, is a signaling mediator intricately linked to pathways involved in cell survival, inflammation, and growth. It is closely associated with critical membrane-bound receptors and serves as a central integration point for multiple stimuli contributing to COPD pathogenesis (Bozinovski et al. 2006). Matrix metalloproteases (MMPs) have been implicated in the progression of emphysema through both direct and indirect mechanisms. Specifically, MMP-9 plays a distinctive role in promoting pulmonary inflammation by breaking down the extracellular matrix, attracting neutrophils to inflammation sites, and amplifying the inflammatory response (Wells et al. 2018).

To understand the molecular mechanisms of *D. spathacea* in treating chronic bronchitis, researchers conducted GO and KEGG enrichment analyses on 66 core genes. The GO analysis revealed that these core genes were significantly associated with various biological processes (BPs), including the cellular response to lipopolysaccharide, peptidyl-tyrosine phosphorylation, negative regulation of the apoptotic process, and positive regulation of MAP kinase activity. The GO function enrichment assessment highlighted processes primarily related to cellular inflammation, proliferation, oxidative stress response, energy metabolism, and apoptosis. This indicates that the response to lipopolysaccharide (LPS) may be the most critical biological process influenced by *D. spathacea* in treating chronic bronchitis.

The KEGG pathway analysis revealed significant associations with pathways such as IL-17, TNF, viral infection, AGE-RAGE, cancer, and PI3K-Akt signaling pathways. Among these, the TNF and IL-17 signaling pathways were notably involved in regulating the inflammatory and immune responses of chronic bronchitis. Notably, three key target genes—PTGS2, MMP-9, and TNF—were involved in both the IL-17 and TNF signaling pathways. These genes, identified as part of the TOP 8 main target genes based on PPI analysis, directly interacted with each other. The TNF and IL-17 signaling pathways play vital roles in regulating inflammatory cytokines, extracellular matrix remodeling, inflammatory mediator synthesis, and pro-inflammatory activities (Bechara et al. 2021; Parameswaran and Patial 2010) (Figs. S11 and S12).

The computational docking process, six key bioactive compounds with three key target proteins, revealed that the formation of hydrogen bonds played a critical role in stabilizing the interactions between the ligands and targets in the complexes (Nguyen et al. 2024). The docking results indicated that the binding affinities of all key bioactive compounds are higher than the reference drug, aspirin. This suggests that key bioactive compounds of *D. spathacea* have potential anti-inflammatory activities, contributing to the therapeutic mechanism of chronic bronchitis. In particular, luteolin showed notable binding affinities across all targets, forming a crucial metal-acceptor interaction with Zn301, which is essential for MMP-9 inhibition. Additionally, luteolin engaged in other interactions, such as hydrogen bonds, and pi-alkyl interactions, contributing to the stability of the complex (Hsu et al. 2021). Salvionoside B showed the lowest docking affinity towards COX-2 (affinity = − 66.5444 kcal/mol), followed by dolichandrone B (affinity = − 65.4134 kcal/mol). Upon analyzing the interaction between proteins and other ligands, it is evident that the extracts of *D. spathacea* exhibit strong binding affinity to the selected targets, achieving lower binding energy primarily through forming multiple hydrogen bonds. This suggests that several key bioactive compounds of this species contribute to the therapeutic effects on chronic bronchitis by targeting key proteins.

Comparison with aspirin validated the ethanol extract’s potential as an anti-inflammatory agent. Notably, at 100 μg/mL, the extract exhibited a comparable or even stronger inhibitory effect on TNF-α (99.155 pg/mL) and IL-1β (2.339 pg/mL) compared to aspirin, highlighting its therapeutic potential in inflammatory conditions. Furthermore, in vitro experiments confirmed that the ethanol extract effectively suppressed the production of nitric oxide (NO) and key cytokines, including TNF-α and IL-1β. These findings suggest that *D. spathacea* may be a promising candidate for treating chronic bronchitis by inhibiting inflammatory responses.

## Conclusions

The study on *D. spathacea* identified three compounds, including the first-time isolation of beta-sitosterol from this species, along with 6-*O*-trans-*p*-coumaroyl ajugol and 6-O-[(*E*)-4-methoxycinnamoyl]catalpol. Network pharmacology and molecular docking analyses showed that key bioactive compounds, such as luteolin, dolichandrone B, salvionoside B, uncaric acid, oleanolic acid, and ursolic acid, effectively bind to proteins linked to chronic bronchitis (such as TNF, MMP-9, and PTGS2) through pathways like TNF and IL-17 signaling pathways. The plant's ethanol extracts showed potent anti-inflammatory effects, notably inhibiting the production of NO, TNF-α, and IL-1β in LPS-stimulated cells. These findings indicate that *D. spathacea* may serve as a promising source of therapeutic agents for addressing chronic bronchitis and inflammatory conditions.

## Supplementary Information

Additional file 1.

## Data Availability

Not applicable.

## Abbreviations

*D. spathacea*: *Dolichandrone spathacea*
CB: Chronic bronchitis
GeneCards: The human gene database
GO: Gene ontology
KEGG: Kyoto encyclopedia of genes and genomes
COPD: Chronic obstructive pulmonary disease
TCM: Traditional Chinese medicine
ADMET: Absorption, distribution, metabolism, excretion, and toxicity
DAVID: Database for annotation, visualization, and integrated discovery
