# Correction: Isolation of phytochemicals and exploration the mechanism of *Dolichandrone spathacea* in the treatment of chronic bronchitis by integrating network pharmacology, molecular docking, and experimental validation

**DOI:** 10.1186/s40529-025-00474-y

**Published:** 2025-08-21

**Authors:** Dang-Khoa Nguyen, Ta-Wei Liu, Man-Hsiu Chu, Quoc-Dung Tran Huynh, Truc-Ly Thi Duong, Thuy-Tien Thi Phan, Duyen Thi My Huynh, Yun-Han Wang, Shu-Mei Wang, Su-Jung Hsu, Ching-Kuo Lee

**Affiliations:** 1 School of Pharmacy, College of Pharmacy, Taipei Medical University, Taipei, 11031 Taiwan; 2 School of Pharmacy, University of Medicine and Pharmacy at Ho Chi Minh City, Ho Chi Minh City, 700000 Vietnam; 3 Program in Clinical Drug Development of Herbal Medicine, College of Pharmacy, Taipei Medical University, Taipei, 11031 Taiwan; 4 Institute of Pharmaceutical Education and Research, Binh Duong University, Binh Duong, 820000 Vietnam; 5 Faculty of Traditional Medicine, Can Tho University of Medicine and Pharmacy, Can Tho, 900000 Vietnam; 6 Graduate Institute of Biomedical Materials and Tissue Engineering, College of Biomedical Engineering, Taipei Medical University, Taipei, 11031 Taiwan; 7 Department of Pharmaceutical and Pharmaceutical Technology, Faculty of Pharmacy, Can Tho University of Medicine and Pharmacy, Can Tho, 900000 Vietnam; 8 Program in Drug Discovery and Development Industry, College of Pharmacy, Taipei Medical University, Taipei, 11031 Taiwan; 9 Institute of Fisheries Science, National Taiwan University, Taipei City, Taiwan

**Correction to: Botanical Studies (2025) 66:24**

10.1186/s40529-025-00464-0

Following publication of the original article (Nguyen et al. 2025), the authors reported that the co-corresponding author designation of Dr. Su-Jung Hsu was missing. She should be listed as a co-corresponding author.

The correct authorship has been provided in this Correction.

The original article (Nguyen et al. 2025) has been corrected.
