# Supplementary material for: Isolation of phytochemicals and exploration the mechanism of Dolichandrone spathacea in the treatment of chronic bronchitis by integrating network pharmacology, molecular docking, and experimental validation
Source: Bot Stud. 2025 Aug 11;66:24. doi: 10.1186/s40529-025-00464-0 (PMC12339790; doi:10.1186/s40529-025-00464-0)
Supplement: Supplementary file 1 — Additional file 1. [file 40529_2025_464_MOESM1_ESM.docx]

**Supplementary material 1**

**Table S1** The genes overlapped between *D. spathacea* and chronic bronchitis

| **Name** | **Elements** |
| --- | --- |
| Overlapped genes between *D. spathacea* and chronic bronchitis | NR3C2, NOS2, MMP3, FGFR1, SERPINA6, ALOX5, TTR, MMP8, CYP2C19, MDM2, EGFR, CD38, CXCR3, PIK3CA, FLT3, ADORA1, IL2, SYK, BCHE, G6PD, EPHX1, PDE5A, ADORA2A, MAPK8, CXCR2, ABL1, CHRM1, CD81, PRKCD, GSTP1, CFTR, SRC, ABCB1, PGR, CHRM2, ATP1A1, ADORA2B, NR3C1, BTK, CHRM4, MAPK14, GC, PDGFRB, TNF, LCK, PLA2G4A, MPO, AKT1, PIK3R1, STAT3, CCR5, F2, PTGS2, CHRM3, PDE4D, MCL1, MMP1, ADA, MMP12, CYP1B1, HSP90AA1, CASP3, MMP9, PARP1, PTPRC, ITGA4 |

**Table S2** Effects of the ethanol extract of *D. spathacea* on NO production, IC₅₀ values, and cell viability in LPS-stimulated RAW 264.7 cells

| **Sample** | **Concentration (µg/mL)** | **Percentage of inhibition** | **IC_50_ (µg/mL)** | **Cell viability (%)** |
| --- | --- | --- | --- | --- |
| Ethanol extract | 400 | 81.10 ± 2.10 | 25.34 ± 0.88 | 80.29 ± 0.65 |
|  | 200 | 91.95 ± 0.61 |  | 90.44 ± 2.63 |
|  | 100 | 86.00 ± 1.64 |  | 97.28 ± 1.54 |
|  | 20 | 40.86 ± 1.19 |  | 98.54 ± 2.75 |
| Dexamethasone | 100 | 81.85 ± 1.48 | 14.18 ± 1.19 | 95.72 ± 2.07 |
|  | 20 | 52.88 ± 1.23 |  | 97.56 ± 1.57 |
|  | 4 | 41.23 ± 1.71 |  | 98.12 ± 1.54 |
|  | 0.8 | 31.56 ± 0.78 |  | 98.76 ± 2.11 |

| **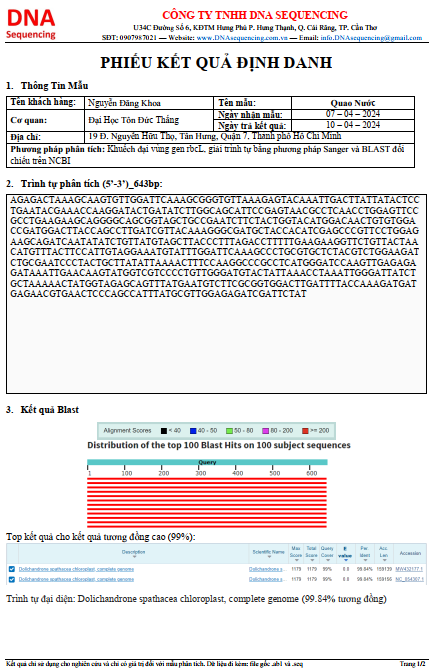** | **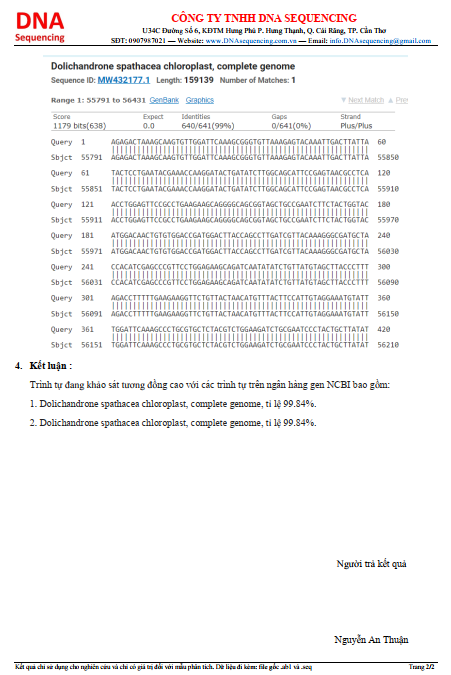** |
| --- | --- |
| **Fig. S1** The identification of *D. spathacea* was conducted using the rbcL gene region method | |


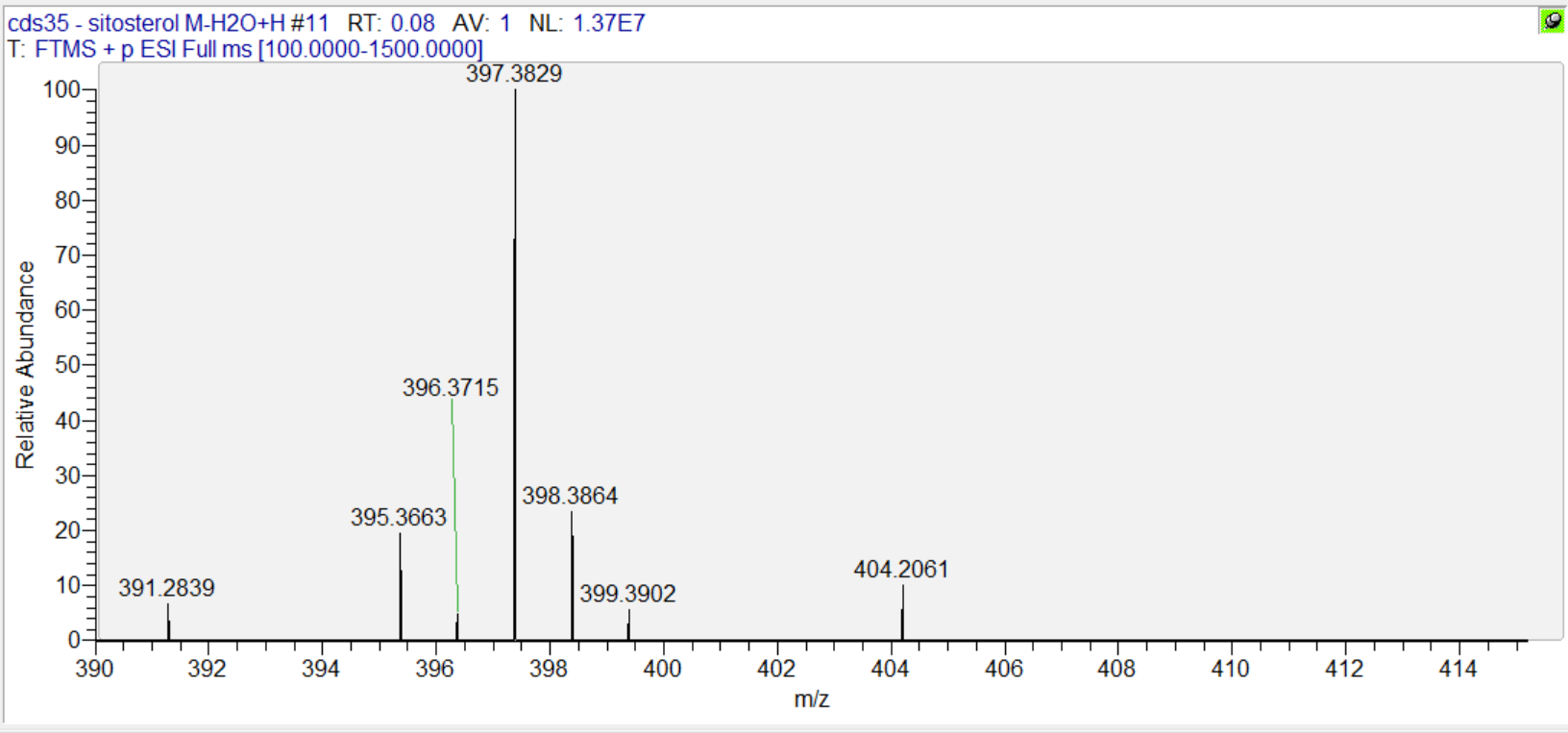


**Fig. S2** HRESI-MS spectrum of beta-sitosterol **(1)**


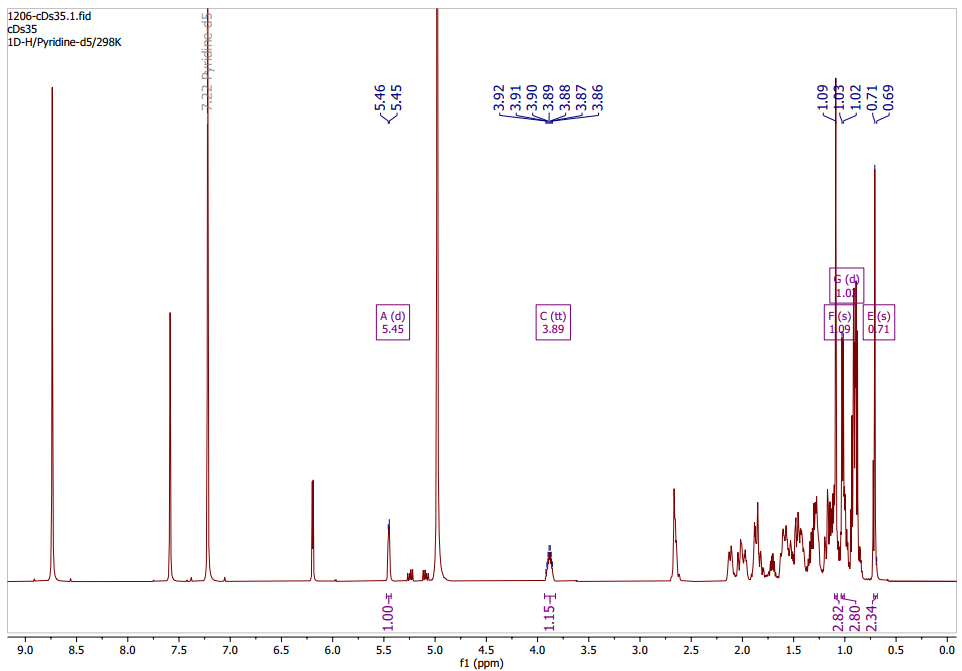


**Fig. S3** ^1^H-NMR spectrum (500 MHz, Pyr-d5) of beta-sitosterol **(1)**

**
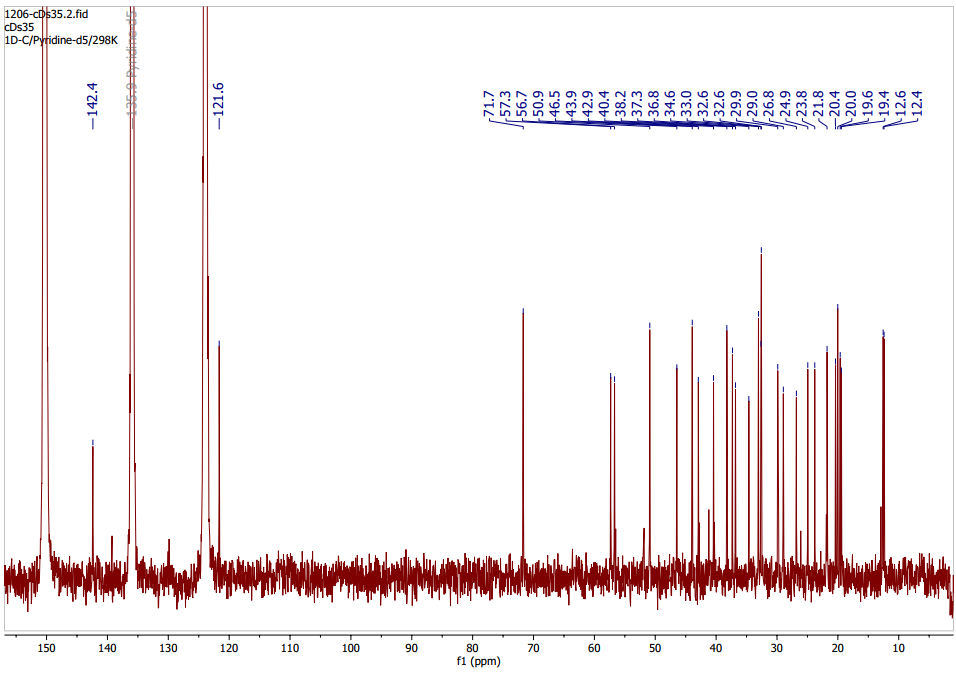
**

**Fig. S4** ^13^C-NMR spectrum (500 MHz, Pyr-d5) of beta-sitosterol **(1)**

**
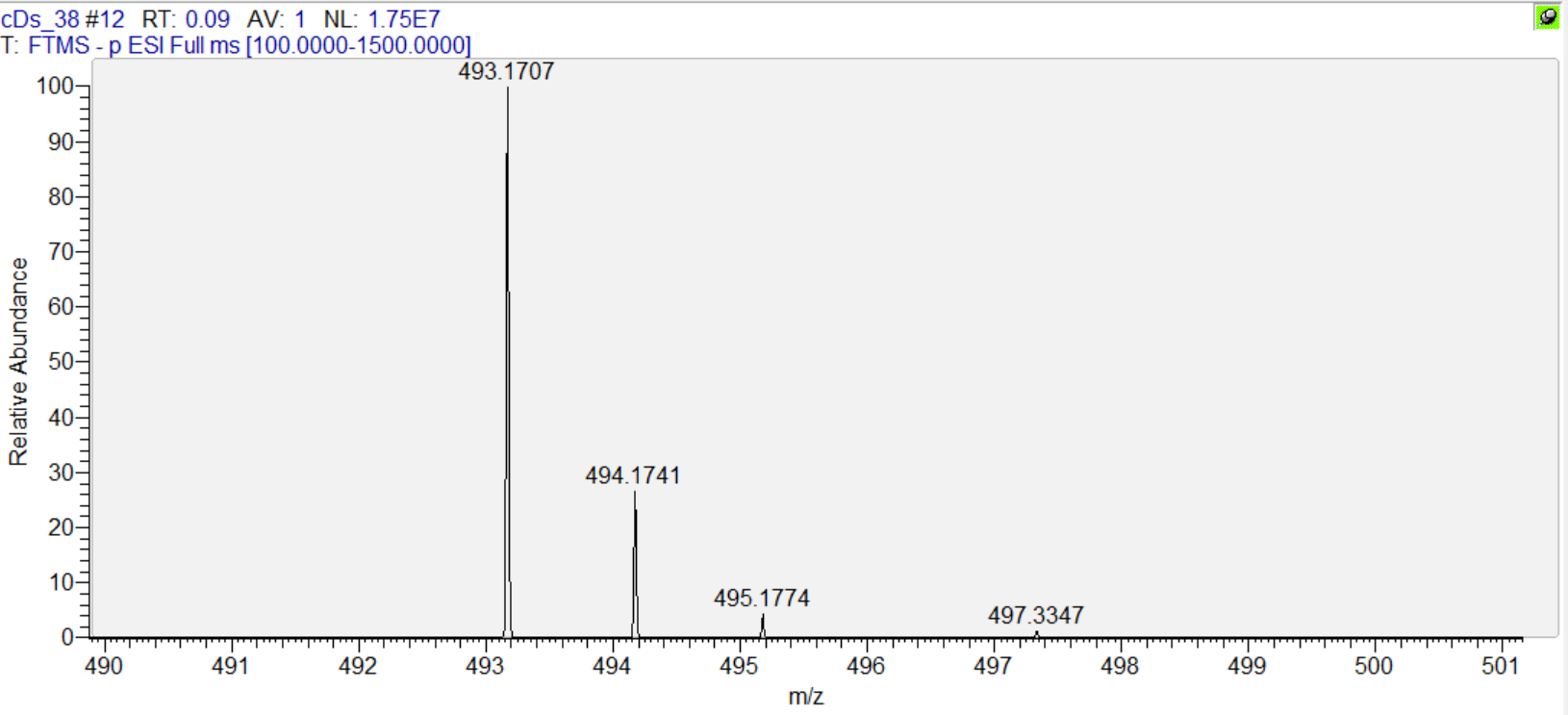
**

**Fig. S5** HRESI-MS spectrum of 6-*O*-*trans*-*p*-coumaroyl ajugol (**2**)


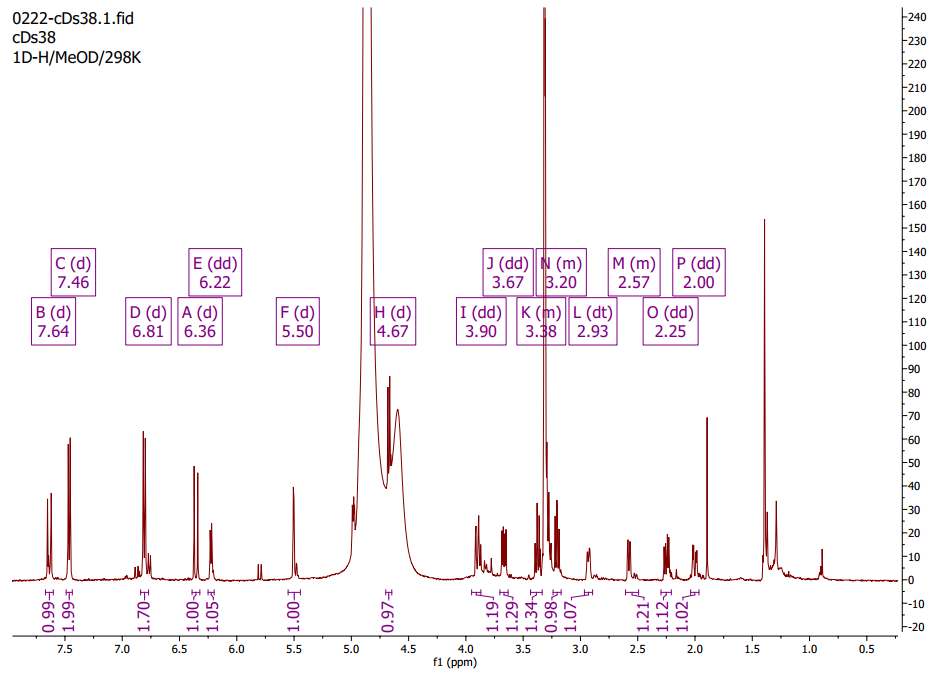


**Fig. S6** ^1^H-NMR spectrum (500 MHz, CD_3_OD) of 6-*O*-*trans*-*p*-coumaroyl ajugol (**2**)

**
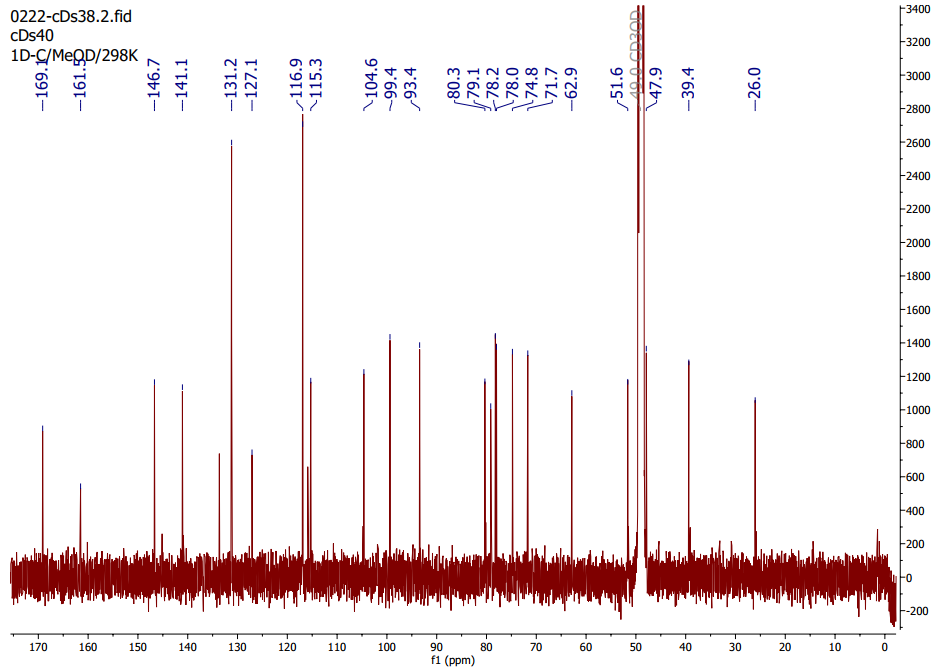
**

**Fig. S7** ^13^C-NMR spectrum (500 MHz, CD_3_OD) of 6-*O*-*trans*-*p*-coumaroyl ajugol (**2**)

**
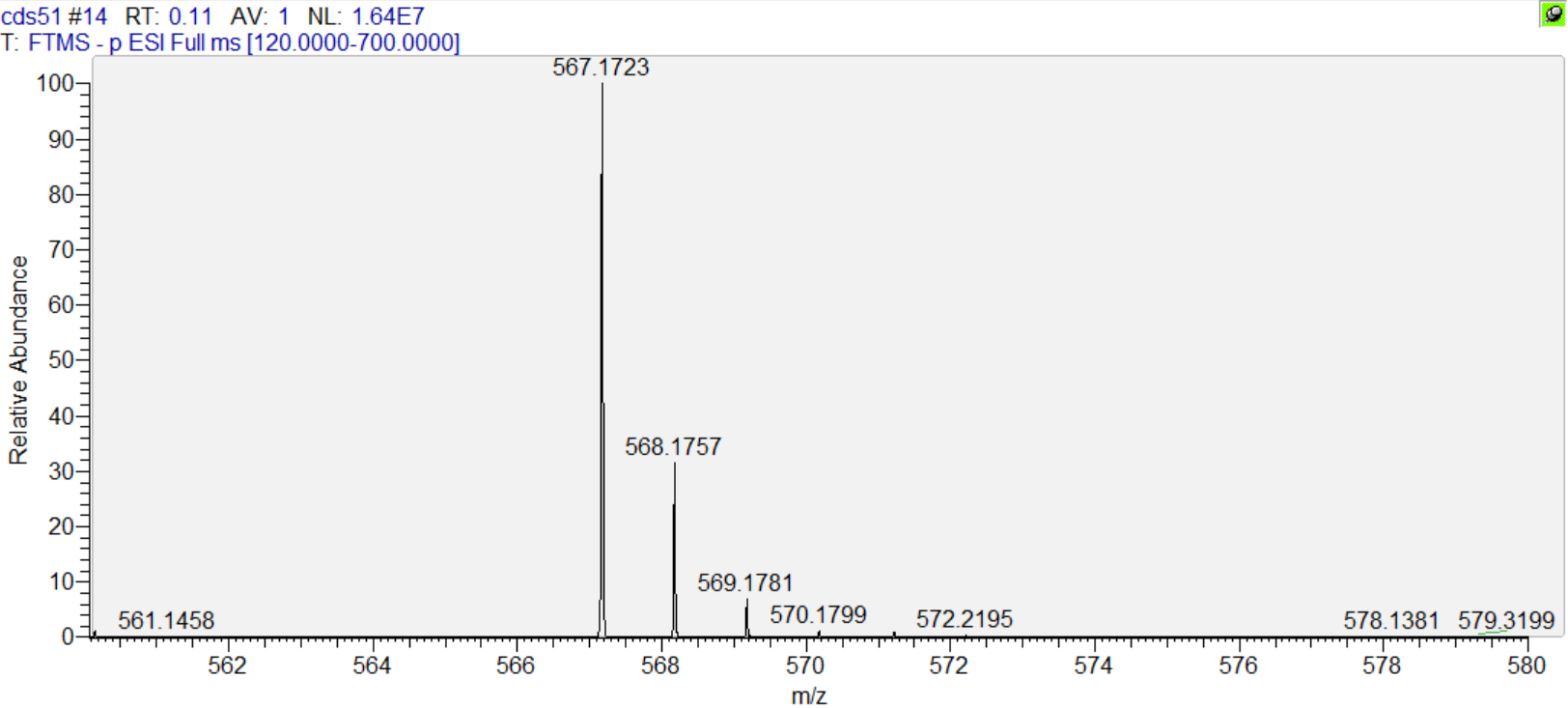
**

**Fig. S8** HRESI-MS spectrum of 6-*O*-[(*E*)-4-methoxycinnamoyl]catalpol (**3**)


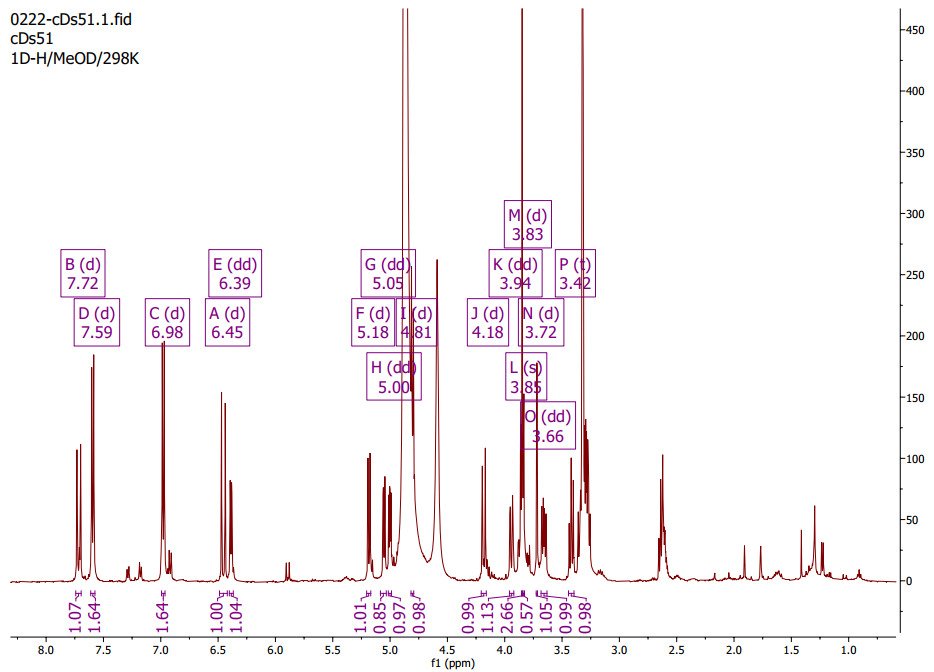


**Fig. S9** ^1^H-NMR spectrum (500 MHz, CD_3_OD) of 6-*O*-[(*E*)-4-methoxycinnamoyl]catalpol (**3**)


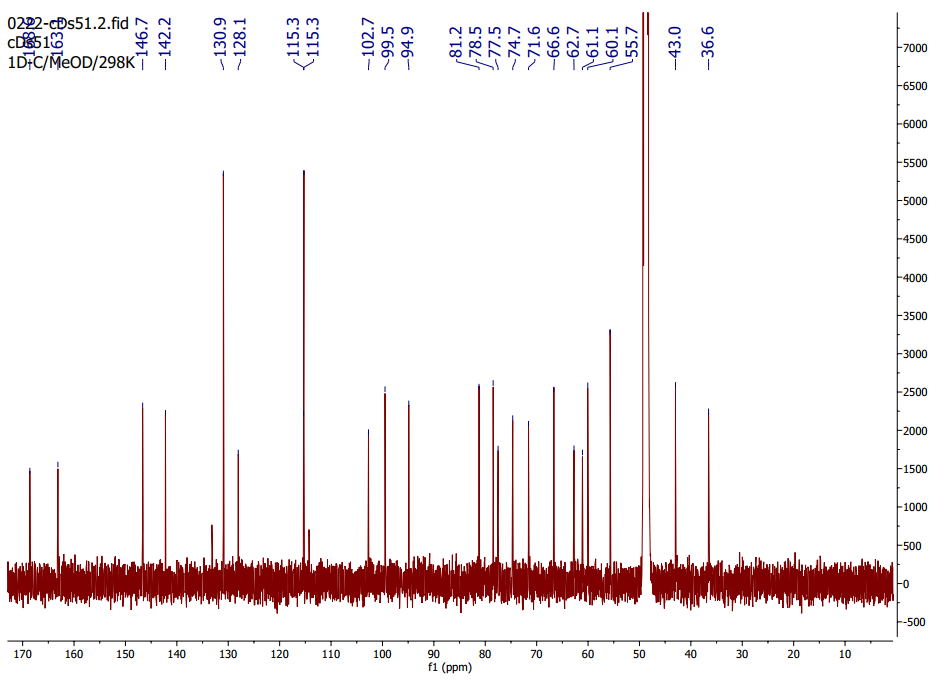


**Fig. S10** ^13^C-NMR spectrum (500 MHz, CD_3_OD) of 6-*O*-[(*E*)-4-methoxycinnamoyl]catalpol (**3**)


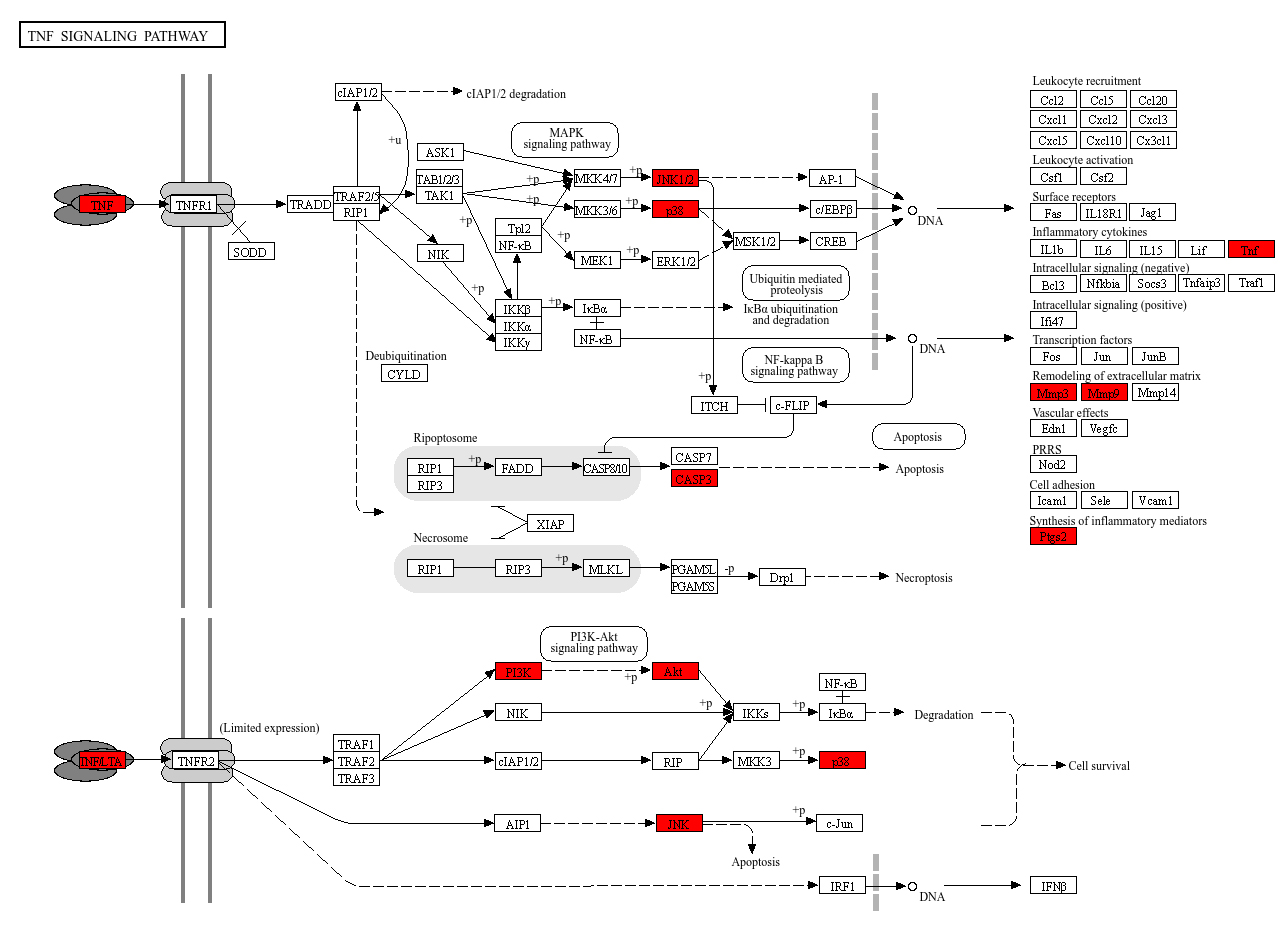


**Fig. S11** The target genes of *D. spathacea* and CB involved in TNF signaling pathway


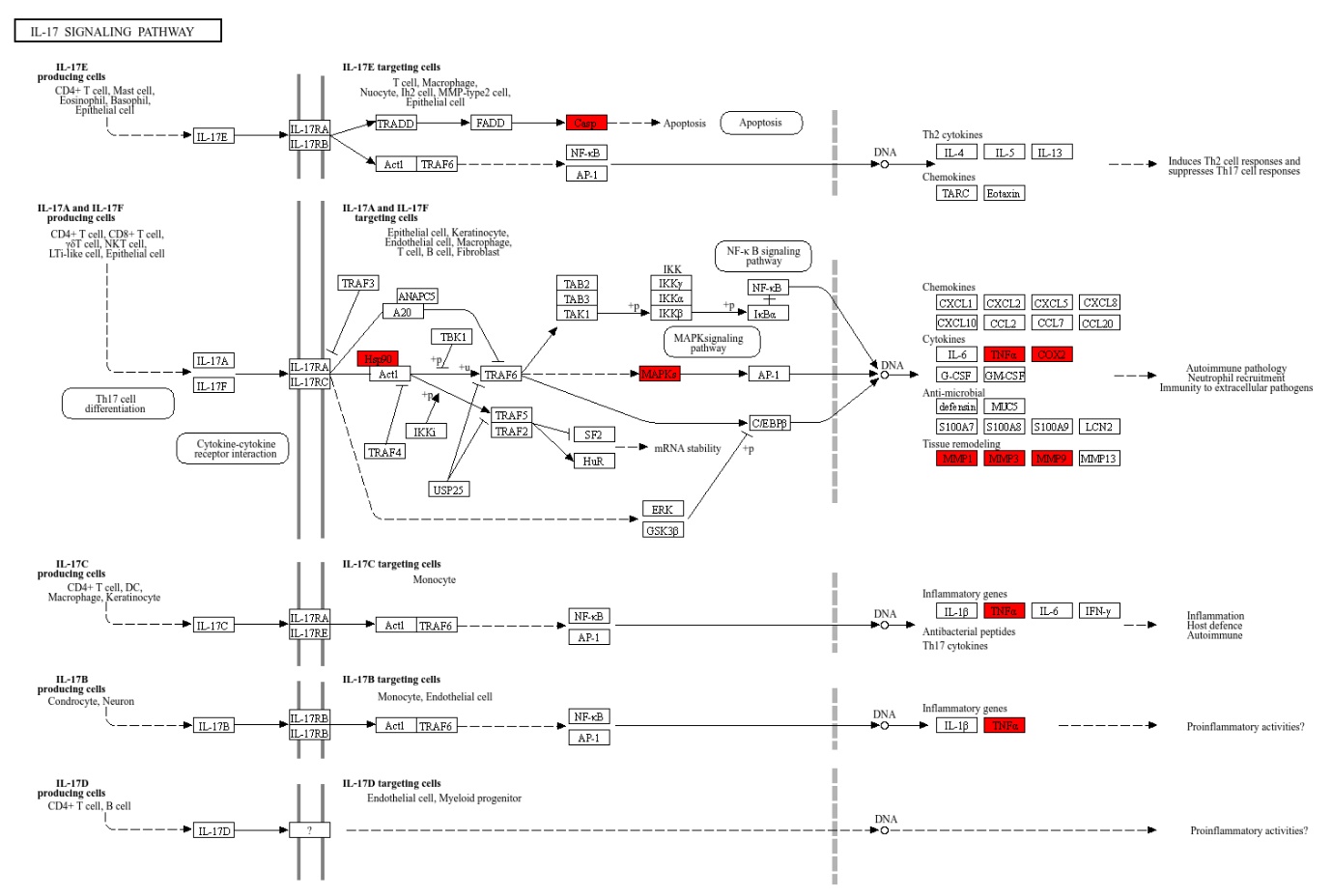


**Fig. S12** The target genes of *D. spathacea* and CB involved in the IL-17 signaling pathway
